# Disruption of Antioxidant Defense Systems in Honey Bees and Wild Bees Under Environmental Xenobiotic Pressure

**DOI:** 10.3390/antiox15081016

**Published:** 2026-08-14

**Authors:** Ivana Tlak Gajger, Josipa Vlainić, Aleksandar Cvetkovikj

**Affiliations:** 1 Department for Biology and Pathology of Fish and Bees, Faculty of Veterinary Medicine, University of Zagreb, 10000 Zagreb, Croatia; 2 Laboratory for Oxidative Stress, Institute Ruđer Bošković, Bijenička Cesta 54, 10000 Zagreb, Croatia; josipa.vlainic@irb.hr; 3 National Veterinary and Food Institute, Faculty of Veterinary Medicine—Skopje, Ss. Cyril and Methodius University in Skopje, 1000 Skopje, North Macedonia; acvetkovic@fvm.ukim.edu.mk

**Keywords:** honey bees, wild bees, antioxidant defense system, oxidative stress, environmental xenobiotics, heavy metals, pesticides, polycyclic aromatic hydrocarbons, microplastic

## Abstract

Honey bee colonies play a vital role in ecosystem stability and global food security. Together with bumble bees and other wild bee species, they form a diverse pollinator community that is particularly vulnerable to environmental pollution. Among stressors, environmental xenobiotics including heavy metals, metalloids, pesticides, polycyclic aromatic hydrocarbons, per- and polyfluoroalkyl substances, and emerging contaminants such as microplastics pose a growing concern due to their persistence, bioaccumulation potential and capacity to trigger oxidative stress and interact with pathogens, nutritional stress and climate-related extremes. The antioxidant defense system, encompassing enzymatic components (superoxide dismutase, catalase, glutathione-dependent enzymes and glutathione-S-transferase) and non-enzymatic antioxidants, represents a key protective mechanism, and its disruption leads to redox imbalance, immunosuppression, and behavioral alterations that can reduce honey bee colony vitality. This review synthesizes current knowledge on the sources, exposure pathways and toxicological effects of major environmental xenobiotics on the antioxidant defense systems of honey bees, with particular emphasis on oxidative-stress biomarkers for early detection of sublethal impairment in field and experimental settings. Where available, evidence from bumble bees and other wild bees is considered to place findings in a broader pollinator-health context and to highlight taxa-specific sensitivities. Work should now concentrate on a validated core panel of redox biomarkers, on chronic multi-stressor exposures that include PFAS and plastic particles, and on biomarker baselines for bumble bees and solitary bees tied to colony- or population-level endpoints.

## 1. Introduction

Honey bees (*Apis mellifera*) are key pollinators of natural and agricultural ecosystems and important bioindicators of environmental conditions, as they integrate spatially diverse chemical contamination across the landscape through their foraging activity [1]. Along with the honey bee, bumble bees and other wild bees form a diverse pollinator community whose pollination services support global food production and biodiversity maintenance, but are also increasingly exposed to the combined action of anthropogenic stressors [2,3]. Globally recorded colony losses and changes in the composition of insect pollinator communities are associated with changes in land use, climatic extremes, pathogens and parasites, and ubiquitous chemical pollution [4,5,6]. In this context, environmental xenobiotics, including pesticides, heavy metals and metalloids, polycyclic aromatic hydrocarbons (PAHs) and emerging contaminants such as microplastics and per- and polyfluoroalkyl substances (PFAS), are increasingly recognized as important drivers of oxidative stress, disruption of homeostasis and increased sensitivity of bees to biological stressors [7,8].

The antioxidant system of bees consists of enzymatic components, among which superoxide dismutase (SOD), catalase (CAT), glutathione peroxidase (GPX) and glutathione-S-transferase (GST) stand out, as well as non-enzymatic antioxidants such as glutathione, ascorbic acid, tocopherols and phenolic compounds from pollen and other plant resources [9,10]. This system functions as a crucial defense against reactive oxygen species (ROS) produced during normal aerobic metabolism and also as a result of exposure to xenobiotics and infections [8]. When exposure is chronic, multiple, or involves synergistic stressors, the antioxidant response may be deficient or dysregulated, leading to the accumulation of oxidative damage to lipids, proteins, and DNA and consequent changes in behavior, immune function, and survival in bees [4,8,11]. Recent work further indicates that dietary antioxidants, particularly polyphenols from pollen and other honey bee products, may modulate redox homeostasis and partially mitigate the effects of environmental stress, which opens up space for targeted nutritional interventions [10,12].

Pesticides are among the most commonly studied environmental xenobiotics in bees and are considered one of the main chemical pressures in intensive agricultural systems [13,14]. Experimental studies show that different classes of pesticides, including neonicotinoids, organophosphates, pyrethroids, and fungicides, can induce increased ROS formation, alter the activity of SOD, CAT, GPX, and GST, and promote lipid peroxidation in honey bees, often at sublethal concentrations with pronounced physiological and behavioral consequences [15,16]. Sub-lethal exposure to neonicotinoids and other pesticides is associated with impairments in cognition, orientation, vitellogenin regulation, and antioxidant capacity, which interact with nutritional stress and heat stress [17,18]. Such disorders in the antioxidant system and redox metabolism are associated with impaired immunocompetence and an increased risk of colony loss, especially in combination with parasites and pathogens [8].

Heavy metals and metalloids, present in soil, water, dust and plant material due to industrial emissions, traffic and agricultural activities, accumulate in adult bees and brood, pollen, wax, other hive products and honey and have been used for decades as indicators of environmental stress [19,20]. Chronic exposure to metals such as cadmium (Cd), lead (Pb), chromium (Cr), copper (Cu), and manganese (Mn) can alter the activity of key antioxidant enzymes and increase oxidative stress, which translates into shortened life expectancy, behavioral changes, and reduced resistance to additional stressors [21]. PAHs, which enter the environment primarily through the combustion of fossil fuels and biomass, have been detected in honey bee products and on plant surfaces, and their lipophilicity favors bioaccumulation and interactions with membrane structures that can mediate oxidative stress and inflammatory responses, including changes in the gut microbiota and the expression of genes related to detoxification [22]. Recent studies using honey bees and hive matrices for PAH biomonitoring show that PAH mixtures associated with airborne particulate matter can affect redox balance and immune-related pathways, while work on diesel exhaust particles in bumble bees indicates concomitant shifts in gut microbiota composition and expression of detoxification- and stress-related genes, highlighting the potential for PAHs and related combustion products to disrupt gut homeostasis and xenobiotic metabolism in pollinators [23].

Microplastics and related adsorbed contaminants represent a new and still insufficiently researched source of pressure on pollinators, but recent research shows that microplastic particles can enter bees through food and water, accumulate in tissues and hive materials, and alter the intestinal barrier, microbiota composition and oxidative status, especially when they occur in combination with pesticides or other chemicals [24]. Experimental work on larvae and adult bees suggests that chronic oral exposure to microplastics and fungicides can alter transcriptomic profiles, antioxidant enzyme activities, and the gut microbiota, with effects that can be complexly modulated [25]. In bumble bees and other wild bee species, data are less abundant compared to those for *A. mellifera*. However, existing studies indicate that their physiological and population responses to a combination of chemical and non-chemical stressors are similar and may even be more pronounced. This could be attributed to their unique life cycles, smaller colony sizes, or solitary habits [26].

Although most of the knowledge on the antioxidant system and oxidative stress has been gathered on the honey bee as a model species, it is increasingly clear that understanding how different environmental xenobiotics individually and in combination impair bees’ antioxidant defenses is key to assessing the long-term consequences for pollinator health and the stability of ecosystem pollination services [8]. This review aims to consolidate the existing knowledge on the sources and routes of exposure to these xenobiotics, their mechanisms of action on oxidative stress and the antioxidant system of honey and wild bees, and to identify key gaps in previous research, with an emphasis on the need for standardized biomarkers and integrative approaches in risk assessment for insect pollinators.

## 2. Literature Search and Study Selection

The data assembled here were retrieved from Scopus, Web of Science Core Collection, PubMed and Google Scholar. Search strings combined a taxon term (*A. mellifera*, honey bee, bumble bee, *Bombus*, *Osmia*, solitary bee, stingless bee, insect pollinator) with a stressor term (pesticide, neonicotinoid, fungicide, herbicide, heavy metal, trace element, polycyclic aromatic hydrocarbon, PFAS, microplastic, nanoplastic, nanomaterial, electromagnetic field) and a redox term (oxidative stress, antioxidant, reactive oxygen species, superoxide dismutase, catalase, glutathione peroxidase, glutathione S-transferase, malondialdehyde, lipid peroxidation, biomarker). The last search was run in June 2026. A record was retained when it reported an antioxidant or oxidative-stress endpoint measured in bees, that is, enzyme activity, gene or protein expression, glutathione status, total antioxidant capacity, or a molecular-damage marker, following defined exposure to an environmental xenobiotic or physical stressor; records quantifying such contaminants in bees or hive matrices were retained where they informed exposure. Studies on the antioxidant properties of bee products as human foods, acute lethality data without a physiological endpoint, and work on non-bee insects were excluded, the last except where cited explicitly as a mechanistic comparator. Priority was given to primary studies and to reviews published from 2020 onwards.

Two features of this evidence base shape everything that follows. Taxonomic coverage is uneven, with *A. mellifera* supplying the great majority of data. The same imbalance has been quantified for contaminant-driven midgut histopathology across aculeate Hymenoptera, where the western honey bee dominated the 74 studies analyzed [27]. Exposure design is the second constraint: most endpoints come from laboratory dosing rather than from apiaries, a limitation also reached by a systematic review confined to pesticides and the honey bee antioxidant system for the period 2020 to 2023 [13]. The present review departs from those syntheses in treating metals, PAHs, PFAS, plastic particles and physical stressors alongside pesticides, and in reading the resulting biomarker patterns as one redox problem rather than as several separate studies. Comparable organ- and biomarker-centered syntheses in other bioindicator taxa show how much interpretive weight this framing can carry [28].

## 3. Antioxidant Defense and Oxidative Stress in Bees

### 3.1. Reactive Oxygen Species and Redox Homeostasis

Bees are exposed to chemical and nutritional stressors that can disrupt cellular redox homeostasis. The main sources of ROS in bees, the enzymatic (SOD, CAT, GPx, GST) and non-enzymatic (glutathione, ascorbate, tocopherols, dietary flavonoids) antioxidant defenses, and the biochemical markers used to monitor oxidative damage and antioxidant response are given in this subsection. Understanding those mechanisms provides a correlation between bee health and ecotoxicological monitoring. However, ROS (superoxide (O_2_^•−^), hydrogen peroxide (H_2_O_2_), and the hydroxyl radical (^•^OH)) are products of cellular aerobic metabolism, which is amplified by specific high energetic demands: flight, activation of immune system against pathogens (e.g., *Varroa destructor* and *Nosema* spp.), and CYP450-mediated biotransformation (pesticides and PAHs); whereas disruption of redox homeostasis arises when ROS production exceeds antioxidant cell capacity causing lipid, proteins, and DNA damage and impairing cellular function [9]. Nutritional diversity (particularly pollen-derived phenolic ingredients) affects antioxidant capacity and xenobiotic tolerance, making redox resilience related to landscape quality.

Mitochondrial respiration is the primary source of ROS, whereas in bees, the high metabolic rate of flight muscle makes this tissue specific in terms of ROS production and possible oxidative damage. Activation of the immune system by pathogen infections triggers NADPH oxidase-driven oxidative processes in hemocytes, whereas chronic infections sustain the production and deplete antioxidant capacities [29]. In addition, CYP450 metabolism (CYP6AS3, CYP9Q1-3) generates superoxide through catalytic uncoupling: PAH biotransformation originates reactive species capable of sustained redox cycling; neonicotinoids impair mitochondrial membrane potential; organophosphates can inhibit antioxidant enzymes; triazole fungicides induce paradoxical pro-oxidant states (induce oxidative stress and impair antioxidant capacities at the same time) [30]. Oxidative stress damages the same molecular targets in bees as in other animals: lipids, through peroxidation and the formation of malondialdehyde (MDA) and 4-hydroxynonenal (4-HNE), which in turn modify proteins and DNA; proteins, through carbonyl adducts that inactivate enzymes; and DNA, through base oxidation [31,32].

### 3.2. Primary Defenses: SOD, CAT, GPx and GST

Superoxide dismutase catalyzes partitioning of superoxide anion radical into hydrogen peroxide and molecular oxygen. CuZnSOD (SOD1) operates in the cytoplasm and MnSOD (SOD2) in mitochondria. Both are upregulated in bees following pesticide exposure, the presence of parasites (especially *V. destructor* mites), and during microsporidia infections (e.g., *Nosema* spp.). Catalase decomposes peroxide to water and oxygen. Glutathione peroxidase reduces peroxide and phospholipid hydroperoxides using GSH as a reductant, whereas the phospholipid-hydroperoxide glutathione peroxidase (PHGPx), expressed in midgut and fat body in bees, is highly structurally and functionally similar to vertebrate GPx4 and thioredoxin systems, providing antioxidant protection within membranes [9]. Glutathione S-transferases are encoded by several genes (~23 genes across five cytosolic classes), and conjugate glutathione to electrophilic substrates, including lipid peroxidation products and xenobiotic reactive metabolites, to render them water-soluble and excretable, having both antioxidant and detoxification roles [33]. Synthesis of its key isoforms (GstD1, GstE1, GstS1) is induced by organophosphates, neonicotinoids, and PAHs via the Nrf2 homolog CncC/Keap1/ARE transcriptional axis [34].

Thus, enzymatic antioxidant systems in the honey bee exhibit complex tissue-specific, developmental, and xenobiotic-induced expression patterns responsible for oxidative stress management, whereas enzymes display pronounced tissue specificity, with the midgut showing high constitutive CAT activity and total antioxidant capacity [35], the fat body demonstrating elevated SOD and CAT expression particularly in the third tergite region, and brain tissue exhibiting specialized antioxidant profiles responsive to neuroprotective interventions, having high SOD but low CAT, relying on GPx and peroxiredoxins for H_2_O_2_ management [36]. Honey bees’ fat body and gut epithelium exhibit the highest antioxidant enzyme activities, reflecting their central detoxification roles [37]. In addition, the bee developmental stage influences enzyme expression. Namely, GST activity increases with time: from larvae through pupae, and GPx peaks in the early larval stage [38]. Xenobiotic exposure induces significant enzymatic modulation: synthetic pyrethroids elevate GST in larvae and pupae [38], and neonicotinoids alter antioxidant defenses in an age-dependent manner with differential susceptibility between queens and foragers. It appears that bee larvae express lower antioxidants and xenobiotic-metabolizing enzyme activities than adults and are more susceptible to oxidative stress. Upregulated CYP450s, GSTs, and antioxidant enzymes due to environmental chemical exposure appear during the nurse-to-forager transition (~day 21 post-emergence) [39]. Sublethal imidacloprid (1 to 10 ppb) upregulates SOD and CAT within 24 to 48 h [29], and organophosphates drive GST induction at concentrations 10 to 100-fold below lethal thresholds [40]. Co-exposure of bees to fungicide and insecticide hinders CYP450 detoxification while still promoting GST induction, providing insights into the complex interactions between xenobiotic and antioxidant transcriptional networks [41]. Queen bees show higher fat body CAT and lower GST than workers, potentially contributing to their multi-year lifespan [42].

### 3.3. Non-Enzymatic Antioxidants and Diet

Glutathione is the most abundant intracellular thiol, competent for directly scavenging ^•^OH and lipid peroxyl radicals and serving as the cofactor for enzymatic reactions by GPx and GST. In healthy cells/tissues, the GSH:GSSG ratio of >100:1 is an indicator of redox status, which is regulated by glutathione reductase (NADPH-dependent) and de novo synthesis via Nrf2/CncC-driven γ-glutamylcysteine synthetase [43]. Ascorbic acid, obtained by bees exclusively from their diet, is another non-enzymatic scavenger of radicals that regenerates α-tocopherol from the tocopheroxyl radical. In addition, alpha tocopherol acquired from pollen lipids is intercalated in membrane phospholipid bilayers and terminates lipid peroxidation chain reactions. These three antioxidants create a recycling network (tocopherol is regenerated by ascorbate, which is regenerated by GSH-dependent enzymes) and thus augment membrane and aqueous-phase protection [44].

Pollen contains flavonols, flavones (luteolin), and cinnamic acids that exert antioxidant effects through radical scavenging, metal chelation, and Nrf2/CncC-mediated upregulation of SOD, CAT and GST; quercetin, for instance, protects honey bees against pesticide- and pathogen-induced oxidative stress in a dose-dependent manner, inducing antioxidant genes at low concentrations and inhibiting CYP450 detoxification at high ones [12]. Moreover, p-coumaric acid induces CYP6AS3, CYP9Q3, and GSTs, functioning as a pivot of bee defense, indicating the importance of dietary phytochemicals in the response of bees to xenobiotic exposure [45]. Vitellogenin, a yolk precursor protein, functions as a critical antioxidant in honey bees, protecting workers from oxidative stress despite their functional sterility [46]. This protein recognizes cellular damage through membrane binding and shields living cells from reactive oxygen species [47]. In solitary insects, vitellogenin mainly facilitates reproduction; in honey bees, it has been repurposed to prolong worker lifespan by reducing oxidative damage, with high titres tracking longevity in both queens and long-lived winter workers. The pattern is best read as a reproductive regulatory pathway remodeled for stress resistance and longevity in a social insect.

### 3.4. Biomarkers of Oxidative Stress: Methods and Tissues

Oxidative stress can produce measurable changes in the hemolymph, fat body, midgut, nervous tissue, and other organs of bees. These responses provide information on physiological adaptation to stress, oxidative injury, and, in some contexts, processes associated with accelerated aging [32]. Table 1 provides a summary of the key biomarkers and the analytical methods commonly used to assess them.

Responses may be evaluated at transcriptional, biochemical, and molecular-damage levels. Frequently examined transcripts include SOD1, SOD2, CAT, GPX, and genes such as gstD1, gstE1, and gstS1, cytochrome P450 genes, thioredoxins, peroxiredoxins, and heat-shock proteins. Transcriptional responses may occur before measurable changes in enzyme activity or oxidative-damage endpoints, although their timing and direction depend on the stressor, dose, tissue, and sampling interval. RNA sequencing allows broader characterization of antioxidants, detoxification, and stress-response pathways, with enrichment analyses frequently identifying glutathione metabolism, xenobiotic processing, and cellular responses to oxidative stress. Metabolomic approaches based on NMR or LC–MS/MS can complement these data by detecting changes in reduced and oxidized glutathione, pyridine nucleotide balance, and intermediary metabolites associated with altered cellular energy metabolism. Integration of transcriptomic, proteomic, and metabolomic data may therefore help distinguish adaptive antioxidant responses from progressive redox dysregulation. Vitellogenin expression or abundance may also serve as an indirect indicator of redox status because of its association with antioxidant capacity, worker longevity, and age-related division of labor.

Oxidative-stress biomarkers are widely used to detect sublethal physiological disruption in honey bees exposed to environmental contaminants and other stressors, sometimes before mortality or colony-level effects become evident [11,48,49]. Common enzymatic indicators include SOD, CAT, GST, GPx and, less frequently, glutathione reductase, whereas non-enzymatic and damage-related endpoints include total antioxidant capacity, the GSH:GSSG ratio, TBARS, protein carbonyls, and oxidatively modified DNA or lipids [11,37,50]. These endpoints are sensitive and applicable in both laboratory and field studies, but their interpretation depends on developmental stage, caste, nutritional status, tissue, exposure duration, and the interval between exposure and sampling [11,37,44].

Biomarker assessment most commonly relies on spectrophotometric enzyme assays performed on whole-body homogenates or dissected tissues, sometimes combined with gene-expression analyses of antioxidant and detoxification pathways [50,51]. Whole-body homogenates are analytically convenient and useful for screening, particularly when sample mass is limited, but they may conceal tissue-specific responses. Analyses of individual tissues are therefore preferable when the biological question concerns a defined exposure route or target organ [32,52]. The midgut is especially relevant in oral-exposure studies because it is a major site of digestion, absorption, and xenobiotic metabolism. CAT and GST activities may differ between the midgut, hemolymph, flight muscle, and other tissues, whereas head homogenates are commonly examined when oxidative endpoints are assessed together with neurotoxicity markers such as acetylcholinesterase activity [51]. Hemolymph permits oxidative markers to be examined alongside immune variables, while abdominal or thorax and abdomen pools provide sufficient material for multi-endpoint biomarker panels in environmental monitoring studies [51,53,54].

Interpretation remains a central difficulty. Increased antioxidant-enzyme activity does not necessarily indicate injury, and it may reflect an adaptive response to increased ROS production. Conversely, reduced activity may result from enzyme inhibition, substrate depletion, diminished synthesis, or failure of antioxidant defense under prolonged or severe stress [44,53]. Damage-related endpoints also require caution. Elevated TBARS or protein carbonyls are compatible with oxidative injury, but their values may vary with diet, age, tissue composition, and physiological state [53]. Biomarker panels are therefore generally more informative than single measurements, particularly when antioxidant-enzyme activities are interpreted together with contaminant concentrations, glutathione status, molecular-damage endpoints, immune variables, behavior, and colony performance [11,51]. Oxidative-stress biomarkers should consequently be regarded as components of an integrated assessment rather than as standalone diagnostic indicators of bee colony health [55].

Taken together, oxidative stress in bees reflects the interaction of metabolic ROS production, immune activation, nutritional status, and xenobiotic biotransformation. Enzymatic defenses are inducible and tissue dependent, while glutathione and dietary phytochemicals provide complementary protection. A combined approach incorporating enzyme activities, the GSH:GSSG ratio, lipid and protein oxidation, DNA-damage endpoints, and transcriptional responses offers a more reliable assessment than any single biomarker. Future studies should establish species- and tissue-specific reference ranges, standardize sampling and analytical procedures, and determine how individual-level redox responses relate to colony performance. Comparable work in bumble bees, solitary bees, and other wild pollinators remains particularly limited, as do studies of chronic multi-stressor exposure and possible transgenerational effects.

## 4. Environmental Xenobiotics and Exposure Pathways

### 4.1. Major Classes of Xenobiotics Relevant to Bees

Bees are exposed to a broad spectrum of environmental xenobiotics that differ in origin, persistence, physicochemical behavior, and toxicological mode of action [56]. The principal contaminant groups of current relevance include pesticides (insecticides, fungicides, herbicides) used in agricultural and urban settings [57]; heavy metals and metalloids originating from industrial activities, traffic and mining; polycyclic aromatic hydrocarbons (PAHs) generated by incomplete combustion (e.g., exposure to smoke used for easier handling of honey bee colonies); per- and polyfluoroalkyl substances (PFAS); and emerging contaminants such as micro- and nanoplastics, engineered nanomaterials and selected pharmaceuticals [1,58,59,60]. Although these classes are often investigated separately, they frequently co-occur in real landscapes and may produce additive, synergistic, or antagonistic biological effects, particularly when exposure is chronic and coincides with nutritional, thermal, or pathogen-related stress [4,61].

Pesticides remain the most extensively documented xenobiotics in bees because residues are commonly detected in nectar, pollen, guttation droplets, surface water and hive matrices, and sublethal exposure has repeatedly been linked to physiological and behavioral impairment [57,62]. By contrast, metals, PAHs and PFAS have long been associated with environmental biomonitoring and spatial pollution mapping, yet there is growing evidence that they also contribute directly to toxic effects in bees and may persist in colonies through contamination of wax, stored food and other hive products [1,59,60,63]. Microplastics and nanoplastics represent an emerging concern not only because they can be ingested or contacted directly, but also because they may act as vectors for co-contaminants, including metals and persistent organic pollutants, thereby modifying effective exposure profiles [64,65].

### 4.2. Environmental Sources and Fate

The environmental sources of bee-relevant xenobiotics are diverse and often overlap spatially. Agricultural practices contribute plant protection products, veterinary medicine residues, fertilizer-associated trace elements, and plastic-derived particles, whereas industrial emissions, mining, waste incineration, vehicular traffic and urban runoff release metals, PAHs, PFAS and complex contaminant mixtures into air, soil and water [2,21,57]. Sewage sludge, landfill leachates and wastewater reuse further broaden the distribution of persistent contaminants and introduce them into agricultural soils and flowering vegetation [66]. Long-range atmospheric transport additionally disperses semi-volatile compounds and particulate-bound contaminants, allowing them to reach regions distant from their source [1,57].

Once released, xenobiotics differ markedly in mobility, persistence and bioavailability. Hydrophilic systemic pesticides can translocate into nectar, pollen and guttation fluids, directly entering bee diets, whereas many PAHs and other lipophilic compounds preferentially partition into waxy plant surfaces, organic matter and beeswax [63,67]. Metals do not degrade and may remain biologically available in soil, dust and water [68], where they can be taken up by plants or ingested during drinking and grooming [21,57]. PFAS are both highly persistent and relatively mobile, enabling their circulation among water bodies, vegetation and biotic matrices [1,69]. Micro- and nanoplastics add further complexity because they can sorb other contaminants on their surfaces, move between terrestrial and aquatic compartments and potentially alter the timing, mixture composition and dose of xenobiotic exposure experienced by pollinators [64,70,71].

### 4.3. Exposure Pathways and Bioaccumulation in Bees

For honey bees, the main exposure pathways are dietary, contact-mediated and in-hive. Nectar and pollen are generally regarded as primary routes for systemic pesticides and other mobile contaminants, while water from puddles, irrigation sources, surface films and guttation droplets can provide additional exposure, especially during warm seasons or brood-rearing peaks [72,73,74]. Honey bees may also contact residues deposited on leaves, flowers, dust particles and soil while foraging, and contaminants adhering to the cuticle or hairs can be transported back to the colony [73]. Honeydew and other plant exudates can represent underappreciated dietary sources of contamination in some landscapes, particularly where atmospheric deposition or plant-surface residues are substantial [1,57].

Within the hive, contaminant circulation continues after foragers return. Beeswax serves as a significant long-term reservoir for various lipophilic xenobiotics, which can accumulate in the wax. These substances can then migrate into brood food, bee bread, and honey, leading to chronic exposure within the hive even when external sources of these chemicals decrease [21,57]. Propolis, stored pollen, and larval food may similarly contribute to repeated internal exposure, especially in colonies located in polluted or intensively managed environments [1]. This internal recirculation is particularly relevant for persistent contaminants because it extends exposure across all developmental stages and castes and increases the likelihood of cumulative and mixture effects [21,64,75].

A substantial biomonitoring literature demonstrates that honey bees and hive products accumulate a range of environmental contaminants and can therefore serve as sentinels of spatial and temporal pollution patterns [1,58,59]. Bees, pollen, wax, propolis and honey have all been shown to contain metals, PAHs, pesticide residues and, more recently, PFAS and microplastics, with contamination profiles often reflecting local land use, industrial pressure and seasonal dynamics [57]. Not all matrices are equally informative: bees and pollen frequently mirror local soil and water contamination more closely than honey, whereas wax is particularly useful for tracking persistent lipophilic compounds [58,59]. These characteristics make honey bees and hive products valuable tools for environmental monitoring. Still, they also highlight the need to consider matrix selection, sampling period, honey bee colony demography and contaminant properties when interpreting biomonitoring and toxicological data [1,57]. Taken together, current evidence indicates that bees experience xenobiotic exposure through multiple, partially overlapping pathways that connect landscape-scale contamination with both external and in-hive accumulation [19,49,73,76]. This complexity underpins the importance of sensitive oxidative stress biomarkers as early-warning indicators of sublethal xenobiotic effects [4], which are addressed in the following section.

## 5. Pesticides and Antioxidant System Disruption

### 5.1. Insecticides (Neonicotinoids, Organophosphates, Pyrethroids and Others)

Insecticides are primarily formulated to target highly conserved neural or metabolic pathways in pest species; however, their application in agricultural environments has pronounced, off-target physiological consequences for both managed and wild bee populations. The induction of oxidative stress has emerged as a central mechanism underpinning the sublethal toxicity of these xenobiotics. Neonicotinoids, including imidacloprid, clothianidin, and thiacloprid, function as potent agonists at nicotinic acetylcholine receptors (nAChRs). This continuous neurostimulation raises cellular energy demands. This, along with mitochondrial oxidative phosphorylation, promotes the leakage of ROS [77,78]. Converging evidence from toxicological assays shows that sublethal exposure overwhelms the antioxidant defense system (ADS): compensatory spikes in SOD and CAT give way to depletion, GST and GPx dynamics are disturbed, and lipid peroxidation follows, with elevated MDA in the affected tissues [78,79,80,81].

Organophosphates, such as coumaphos, and pyrethroids trigger similar oxidative pathologies [81,82]. For instance, acute sublethal exposure to the pyrethroid deltamethrin impairs the redox equilibrium within the honey bee central nervous system, depressing the GSH/GSSG ratio and elevating MDA levels, even when hydrogen peroxide concentrations appear stable, indicating an incomplete and failing compensatory response [17]. Similarly, exposure to lambda-cyhalothrin downregulates SOD in developing larvae and subsequently induces midgut cell damage in adults, despite transient early spikes in CAT activity [83,84]. The damage extends beyond the exposed life stage: chronic larval ingestion of lambda-cyhalothrin reshapes the expression of antioxidant and neural-signaling genes in ways that persist into adulthood, generating a developmental legacy of physiological fragility in newly emerged workers [85].

This pesticide-induced redox disruption is associated with functional deficits across the organism. In the brain, oxidative damage directly correlates with degraded cognitive architecture, leading to pronounced deficits in olfactory learning, associative memory (frequently measured via proboscis extension reflex assays), and spatial orientation during critical homing flights [17,77,86]. The metabolic reallocation needed to sustain high detoxification and antioxidant enzyme activity also imposes an energetic drain. This state of physiological exhaustion strongly suppresses transcription of vitellogenin, a pleiotropic glycolipoprotein that is fundamental to extending worker lifespan and buffering cellular oxidative damage [8,43,87]. With vitellogenin depleted and the glutathione system compromised, xenobiotic-stressed bees exhibit marked immunosuppression. A consensus of field and laboratory studies indicates that this compromised state heavily suppresses the Toll and Imd immune signaling pathways, significantly increasing the replication rates and virulence of opportunistic pathogens like the gut microsporidian *Nosema ceranae* and Deformed Wing Virus (DWV) [8,77,79,88].

### 5.2. Fungicides and Herbicides

Historically classified as “bee-safe” due to their relatively high acute lethal dose (LD50) thresholds, fungicides and herbicides represent a pervasive and underappreciated threat to pollinator redox homeostasis. Because these compounds are routinely applied to crops during full bloom, foraging bees experience chronic, high-volume exposure that provokes substantial sublethal metabolic stress. Triazole fungicides, primarily used as sterol biosynthesis inhibitors (SBIs) to control fungal pathogens, exhibit significant off-target toxicity to bees. Chronic ingestion of SBIs markedly disrupts redox balance in high-demand tissues, including flight muscles. While bees often mount a vigorous compensatory response, upregulating total antioxidant capacity and glutathione activity to stabilize ROS, the metabolic cost of this adaptation is steep and likely accelerates physiological senescence [89]. Broad-spectrum herbicides similarly disrupt the oxidative balance. Glyphosate, which targets the shikimate pathway in plants, induces significant mitochondrial dysfunction in honey bees. Following sublethal exposure, bees exhibit an immediate upregulation of antioxidant and mitochondrial-related genes; however, within 72 h, this transcriptional effort routinely fails to prevent a significant increase in lipid peroxidation adducts, confirming an uncompensated state of oxidative damage [90]. The oxidative footprint of glyphosate is amplified indirectly through the gut microbiota, which contributes substantively to host antioxidant and immune homeostasis, herbicide-driven dysbiosis therefore prolongs the window during which the bee remains vulnerable to ROS-mediated injury [91]. The principal pesticide classes, their molecular targets, reported oxidative-stress biomarkers, and associated antioxidant/redox responses are summarized in Table 2.

The most critical risk associated with fungicides lies not in their isolated toxicity, but in their capacity to drive synergistic interactions when co-applied with insecticides. EBI fungicides are potent competitive inhibitors of cytochrome P450 monooxygenases (CYPs), the primary enzyme superfamily responsible for Phase I xenobiotic detoxification in insects [92,94]. By binding to the catalytic sites of critical bee CYP isoforms, triazoles impair the bee’s ability to metabolize and excrete concurrent insecticidal contaminants. Extensive toxicological data demonstrate that co-exposure to SBIs (such as propiconazole or prochloraz) and pyrethroids (such as tau-fluvalinate) or cyano-substituted neonicotinoids (such as thiacloprid) markedly increases the acute toxicity and oxidative damage of the insecticide, sometimes by several hundred-fold [79,92]. This biochemical synergy transforms otherwise manageable, low-dose insecticide exposures into highly debilitating oxidative events. Mechanistic precision matters here. The synergy maps largely onto inhibition of the CYP9Q subfamily, which encodes the principal isoforms responsible for metabolizing both pyrethroids and N-cyanoamidine neonicotinoids in *A. mellifera*; loss of CYP9Q function therefore translates directly into elevated systemic exposure to the parent insecticide [93,95]. The main functional consequences of pesticide-induced oxidative stress and the environmental factors that modify these responses are summarized in Table 3.

### 5.3. Mixtures and Field-Realistic Co-Exposures

In contemporary agroecosystems, pollinators rarely encounter single active ingredients. The “in-hive exposome” comprises a highly complex, fluctuating matrix of insecticides, fungicides, herbicides, and acaricides [62,80]. Evaluating the oxidative outcomes of these chronic, low-dose, multi-pesticide co-exposures is critical for understanding realistic colony declines. Experimental administration of field-realistic chemical cocktails, such as binary or ternary mixtures of imidacloprid, difenoconazole, and glyphosate, reveals considerable systemic redox disruption across the head, midgut, and abdominal tissues of winter honey bees [80,96].

The toxicodynamics of these mixtures are highly unpredictable and do not uniformly conform to simple synergistic models. Depending on precise concentrations and the specific combination of molecules, responses can be additive, synergistic, or distinctly antagonistic, driven by the complex, simultaneous induction or inhibition of specific CYP and GST isoforms [80]. Irrespective of the exact interaction geometry, the cumulative burden of detoxifying a chemical cocktail rapidly exhausts the bee’s GSH reserves and overwhelms CAT and SOD capacities. The result is widespread protein carbonylation, lipid peroxidation, higher mortality and reduced colony fitness [80,97,104]. Nutritional context sets the severity of these outcomes: polyphenol-rich polyfloral pollen buffers pesticide-induced oxidative injury, whereas monoculture forage withholds that buffer and leaves colonies sensitive to trace agrochemical residues [12,62,105]; the mechanisms are treated in the section on nutritional modulation below. Thermal stress driven by climatic extremes forces bees to increase metabolic thermoregulation, thereby elevating baseline endogenous ROS production and compounding the overall oxidative burden on the failing detoxification system [103,106]. The thermal–chemical relationship is not unidirectional. Sublethal pesticide exposure itself impairs the workers’ capacity to maintain colony-level thermoregulation, generating a positive feedback loop in which xenobiotic burden and thermal dysregulation reinforce one another [93]. The cocktail also extends beyond agrochemicals: Cd and lead, leached into nectar and pollen from anthropogenic point sources, interact additively with neonicotinoids and herbicides to depress survival and impair appetitive learning further [102,103,107].

While the mechanistic links between xenobiotics and oxidative stress are becoming clearer, significant limitations in the current literature preclude the establishment of universal toxicological models. Current datasets are highly fragmented by variable experimental designs, employing vastly different exposure regimes (acute topical versus chronic dietary) and analyzing disparate endpoints (whole-body homogenates versus isolated midgut or brain tissues) [92]. This variance makes cross-study comparison difficult. Many toxicological assessments also rely heavily on the western honey bee (*A. mellifera*) as a singular model organism. A systematic review of pesticide effects on the honey bee antioxidant system covering 2020 to 2023 reached the same conclusion [13]. This approach fails to capture the unique physiological sensitivities and distinct detoxification capacities of diverse wild bee species, such as *Bombus* and *Osmia*, which frequently exhibit different metabolic responses to pesticide mixtures [95,108]. A transition from short-term LD50 testing toward the longitudinal assessment of physiological biomarkers under field-realistic, multi-stressor conditions is essential to accurately characterize the environmental risks imposed on global pollinator populations [109,110].

## 6. Metals and Metalloids

### 6.1. Environmental Occurrence and Bioaccumulation in Bees

Trace metal elements and metalloids, including Cd, Pb, mercury (Hg), arsenic (As), and Cu, constitute a persistent and escalating threat to global pollinator populations. Unlike many synthetic organic agrochemicals, heavy metals resist environmental degradation and continuously cycle through soil, water, and atmospheric compartments [7,111]. Due to their extensive foraging range, regularly covering areas up to 7 km^2^, honey bees act as highly efficient, active biological samplers of these persistent contaminants [55,112]. Foragers intercept heavy metals through multiple exposure routes: they ingest plant-translocated metals in contaminated nectar and pollen, and they passively collect airborne particulate matter (PM10 and PM2.5) from industrial emissions or vehicle exhaust directly onto their branched cuticular setae [113,114,115]. Among workers, older foragers are the first to encounter contaminated resources and typically carry the highest body burdens of toxic elements [1].

Once introduced into the honey bee colony, these metals partition unevenly across different hive matrices, a phenomenon well documented by convergent evidence from numerous biomonitoring surveys. Beeswax, primarily composed of lipophilic substances, functions as a long-term reservoir because combs are routinely reused across successive generations, wax continuously accumulates airborne particulates and lipophilic contaminants, occasionally reaching toxicologically relevant concentrations [59,76,116,117,118]. Pollen and propolis similarly exhibit high metal burdens that closely mirror the surrounding environmental pollution levels [119,120,121]. Comparative biomonitoring surveys in Croatia and Italy have used these matrix-specific metal profiles to distinguish urban-industrial zones from agricultural regions, illustrating the sensitivity of bees as sentinel species [49,122]. By contrast, honey consistently carries markedly lower heavy metal concentrations [119,123].

This striking disparity does not imply a lack of environmental exposure but rather points to an active endogenous biofiltration mechanism within the bees themselves. As foragers process nectar in their proventriculus and midgut, heavy metals are actively sequestered into specialized intracellular granules within the fat body and midgut epithelium, or they are directly excreted in feces [123,124]. While this physiological filtering effectively buffers the colony’s central carbohydrate reserve from pronounced contamination, it imposes a considerable metabolic and toxicological burden directly onto the individual worker bee, where cellular damage accumulates [123,125].

### 6.2. Mechanisms of ROS Induction and Antioxidant Inhibition

The primary cytotoxic mechanism of heavy metals involves marked disruption of cellular redox homeostasis. Toxic metals bypass normal metabolic controls to induce oxidative stress, utilizing two distinct biochemical pathways depending on their specific valency and oxidative properties. Redox-active transition metals, such as Cu and Fe, directly catalyze the production of hydroxyl radicals from hydrogen peroxide via Fenton and Haber-Weiss chemistry [103,126,127]. Non-redox-active metals, such as Cd and Pb, generate ROS indirectly. These elements possess exceptionally high thiophilicity, allowing them to bind irreversibly to the essential sulfhydryl (-SH) groups of structural proteins and enzymes [127,128]. This action directly neutralizes primary non-enzymatic antioxidants, particularly reduced GSH, and inactivates thiol-dependent defense proteins, shifting the cellular environment toward oxidative imbalance [127]. Both pathways culminate in substantial mitochondrial dysfunction, the primary site of cellular respiration, leading to electron transport chain decoupling and sustained superoxide anion leakage [84].

Experimental evidence indicates that the honey bee ADS exhibits a complex, dose-dependent response to chronic metal exposure. Sublethal oral ingestion of Cu or Cd initially triggers a strong, compensatory transcriptional upregulation of SOD1 and CAT genes in the bees’ midgut and flight muscles [127,129]. However, this transcriptomic effort frequently fails to translate into effective enzymatic protection. Actual CAT and SOD enzyme activities often remain static or significantly decrease under heavy metal stress, likely due to direct molecular inhibition by Pb or enzymatic degradation by the sustained surge of free radicals [127,130]. As primary defenses fail, researchers consistently record elevated levels of MDA, a well-established biomarker of unchecked lipid peroxidation that signifies structural damage to cellular membranes [81,131,132]. An essential secondary defense mechanism against Cd toxicity is the induction of metallothioneins (MTs), low-molecular-weight and cysteine-rich proteins that sequester free metal ions to prevent further ROS generation [129,133,134]. While acute Cd exposure reliably increases MT expression, evolutionary constraints sharply limit this adaptation. Genomic consensus establishes that honey bees possess a distinctly contracted detoxification repertoire, and they encode only a single MT gene and harbor approximately half the GST and cytochrome P450 loci found in other insect models like *Drosophila melanogaster* [129,130]. This inherent genetic bottleneck renders the honey bee particularly vulnerable to uncompensated oxidative stress, as its baseline detoxification machinery is easily saturated by chronic heavy metal exposure [129,130].

### 6.3. Sublethal Effects and Interaction with Other Stressors

When intrinsic antioxidant defenses collapse under metal-induced pressure, the ensuing cellular damage drives a cascade of considerable sublethal behavioral and physiological deficits. Neural tissues are acutely susceptible to oxidative injury owing to their intense metabolic oxygen demand and lipid-rich membrane architecture. The accumulation of ROS and subsequent lipid peroxidation physically degrades synaptic integrity, directly impairing cognitive architecture [81,132]. Empirical data derived from proboscis extension response (PER) conditioning paradigms demonstrate that sublethal body burdens of Cd, Pb, and As degrade associative learning acquisition and reduce long-term olfactory memory specificity [86,102]. This cognitive erosion prevents foragers from efficiently associating floral scents with nutritional rewards, degrading foraging performance and homing success [86,102]. Compounding this neurological injury is a sensory blind spot where electrophysiological and behavioral assays confirm that honey bees cannot detect or actively reject these toxic metals at field-realistic concentrations in nectar, permitting continuous, unrecognized ingestion until physiological failure occurs [57,135].

Immune function suffers parallel degradation under metal exposure. The metabolic reallocation required to sustain continuous detoxification, coupled with direct oxidative damage to circulating hemocytes, suppresses the bee’s innate immune system [134]. Chronic metal exposure inhibits critical phenol oxidase activity and reduces the transcription of antimicrobial peptides such as abaecin and defensin [79,136,137]. This immunocompromised state strips the honey bee colony of its primary defense mechanisms, allowing the proliferation of opportunistic gut pathogens like *N. ceranae* and viral infections [120,134,138]. Mechanistically, oxidative damage to the peritrophic membrane and midgut epithelium itself appears to lower the colonization threshold for these parasites, intensifying the metabolic burden through a chemical-biological feedback loop [120]. A quantitative review of contaminant-driven midgut histopathology in bees and other aculeate Hymenoptera confirms that epithelial degradation and peritrophic membrane damage are the commonest lesions recorded, and that insecticides and fungicides account for most of the tested exposures [27].

In modern agroecosystems, solitary and managed bees rarely encounter single contaminants. The environmental exposome features complex, fluctuating matrices of metals, synthetic pesticides, and nutritional deficits. Converging data from advanced co-exposure models reveal that combinations of metals, such as Cd and Cu, provoke synergistic toxicity, reducing larval development rates, depressing adult sucrose consumption, and accelerating mortality far beyond simple additive predictions [86,139].

When heavy metals intersect with agricultural chemicals, the combined oxidative burden frequently overwhelms the organism. For instance, concurrent exposure to Pb and the widely applied herbicide glyphosate, demonstrated in *A. cerana*, but biologically plausible across *Apis* spp., synergistically impairs memory retention and depresses GST detoxification pathways, limiting the bee’s capacity to clear the chemical load [137]. Mixtures of herbicides and Cd similarly disrupt retinoid metabolism and deplete essential dietary carotenoids, exhausting the bee’s non-enzymatic antioxidant reserves [103]. Current toxicological models that rely on isolated, single-chemical LD50 testing systematically underestimate these interactive threats [86]. To accurately safeguard pollinator populations, the available evidence supports a shift toward evaluating the synergistic, oxidative impacts of these persistent, multi-stressor chemical networks [86,137]. At the colony scale, energy diverted from foraging and brood care toward this defense response contributes to premature aging of the worker population and a chronic erosion of demographic resilience [77].

## 7. Polycyclic Aromatic Hydrocarbons

### 7.1. Sources, Occurrence and Bioaccumulation

Polycyclic aromatic hydrocarbons (PAHs) are persistent, widely distributed organic pollutants generated primarily through the incomplete combustion of carbonaceous materials [140]. In contemporary foraging environments, honey bee colonies are chronically exposed to these toxicants through multiple sources of emissions. Converging environmental monitoring data identify vehicular traffic exhaust, industrial emissions from petrochemical and cement facilities, and seasonal biomass burning as the primary drivers of ambient PAH loads [7,141,142]. Domestic heating, particularly coal and wood combustion, further exacerbates this pollution, predictably shifting the environmental profile toward elevated concentrations of low-molecular-weight, three-ring PAHs during the autumn and winter months [141,143]. Because foraging honey bees regularly traverse huge areas, their densely branched cuticular setae act as highly efficient, active bio-samplers of airborne particulate matter, effectively sweeping PAHs from the atmosphere [144,145].

Upon return to the hive, these hydrocarbons partition unevenly across different in-hive matrices, governed strictly by the physicochemical properties of the specific compounds and the receiving matrix. Consensus across systematic reviews and extensive field sampling indicates that honey consistently has the lowest overall PAH burden [145,146]. Due to its high aqueous and sugar content, honey strongly repels highly lipophilic, high-molecular-weight (HMW) PAHs, sequestering primarily low-molecular-weight (LMW) compounds such as naphthalene, phenanthrene, and fluorene [140]. While benzo[a]pyrene (BaP) remains the prioritized toxicological marker for regulatory monitoring, it is these LMW hydrocarbons that frequently drive peak quantitative concentrations within the nectar-derived matrix [146]. Beyond setae-mediated capture, the electrostatic charge that bee bodies acquire during flight further drives adsorption of airborne particulates and their associated PAHs [113]. Within the honey matrix specifically, phenanthrene typically dominates the LMW profile in fresh samples even from areas distant from major industrial centers, consistent with widespread atmospheric deposition as the principal contamination pathway [140]. By contrast, pollen and propolis show strong bioaccumulation of heavier, highly mutagenic PAHs. Pollen features a lipid-rich pollen coat (comprising up to 22% lipids depending on the floral origin), while propolis is inherently resinous, and both matrices act as powerful lipophilic sinks [145,147]. Consequently, pollen and propolis frequently contain elevated levels of four- to six-ring PAHs, including fluoranthene, chrysene, and BaP, reliably mirroring the contamination profile of the surrounding industrial or urban soil and air [22,141].

Foraging bees themselves maintain high systemic PAH concentrations, providing a highly representative snapshot of recent atmospheric exposure, though analysts must apply measured skepticism when interpreting isolated LMW PAH spikes in hive matrices, as the routine use of combustion smokers by beekeepers can introduce localized, artifactual contamination [144,145,148], especially in combs. Ultimately, the strategic selection of the sampling matrix, preferencing pollen or the honey bees themselves over honey, is essential for an accurate toxicological assessment of environmental PAH loads [145].

### 7.2. Metabolism, CYP450 Activation and ROS Generation

Unlike heavy metals, which directly induce cytotoxicity, PAHs are relatively inert upon initial ingestion or cuticular absorption, and their pronounced physiological threat depends entirely on host-mediated metabolic bioactivation. To clear lipophilic xenobiotics, insects employ Phase I detoxification mechanisms, primarily relying on the cytochrome P450 monooxygenase (CYP450) superfamily [149]. Specific aryl hydrocarbon hydroxylases in this enzymatic class hydroxylate PAHs, increasing their hydrophilicity for subsequent excretion [149,150]. However, this biotransformation paradoxically converts parent PAHs into highly electrophilic epoxides, radical cations, and dihydrodiols [140,149]. These reactive intermediates are markedly cytotoxic, readily forming covalent adducts with nucleophilic sites on cellular DNA and structural proteins, thereby driving mutagenesis and substantially disrupting cellular architecture [140].

The metabolic activation of PAHs inherently disrupts cellular redox homeostasis. The continuous cycling of CYP450 enzymes during intense PAH exposure requires substantial electron transfer, often leading to electron leakage and the production of large amounts of ROS. Evidence derived from isolated aquatic insect models, such as mosquito larvae, clearly demonstrates that exposure to environmentally relevant concentrations of fluoranthene and BaP directly catalyzes dose-dependent surges in superoxide anion formation [132]. Crucially, this ROS generation is significantly amplified under UV-A photoactivation, a factor highly relevant for diurnal pollinators continuously exposed to solar radiation during foraging flights [132].

While direct, laboratory-controlled toxicokinetic dosing of single PAHs in *A. mellifera* remains methodologically limited, field data extrapolated from bees foraging in heavily anthropized, PAH-contaminated environments confirm a pronounced alteration of the intrinsic ADS. When exposed to complex urban air pollution heavily burdened with PAHs, honey bees exhibit distinct, compensatory fluctuations in primary antioxidant enzymes [136,151]. SOD and CAT activities are frequently altered as the organism attempts to quench the surging intracellular hydrogen peroxide and superoxide levels [130,136]. Concurrently, GST, the critical Phase II enzyme responsible for conjugating reduced glutathione to PAH-epoxides to neutralize their reactivity, is heavily mobilized [136]. The evolutionary reality that honey bees possess a markedly contracted detoxification genome, harboring roughly half the CYP450 and GST genes of other model dipterans, renders this metabolic pathway exceptionally fragile [130,150]. When the limited GST and glutathione reserves are exhausted by continuous PAH bioactivation, unchecked ROS rapidly precipitate lipid peroxidation, marked by elevated MDA adducts in the insect’s midgut and neural tissues [84,136].

Beyond lipid peroxidation, the reactive intermediates generated during PAH bioactivation extend their damage to nucleic acids. The canonical activated metabolite of BaP (benzo[a]pyrene-7,8-dihydrodiol-9,10-epoxide) readily forms covalent adducts with guanine residues, producing the bulky DNA lesions long established as drivers of mutagenesis in mammalian and dipteran models [149]. Emerging data in *Apis* spp. suggest analogous pathways operate, like sublethal exposure to oxidative stressors triggers nuclear DNA fragmentation and apoptotic signaling within the midgut epithelium and brain [152,153]. These cellular insults carry behavioral consequences that matter at the colony scale, including measurable deficits in olfactory learning, spatial navigation, and foraging efficiency [154], all of which feed back into the longevity and resilience deficits described above.

### 7.3. PAHs in Mixtures and Interaction with Other Pollutants

In authentic agricultural and urban ecosystems, pollinators do not encounter PAHs as isolated chemical entities. The modern environmental exposome dictates that PAHs co-occur within a highly complex, fluctuating matrix of inorganic pollutants and synthetic agrochemicals. Vehicle-derived ultrafine particulate matter (PM2.5) serves as a primary physical carrier in these environments, arriving at the hive coated simultaneously with pyrogenic PAHs, catalytic transition metals (such as Fe, Cu, and Pb), and trace pesticide residues [86,155]. Evaluating the toxicological outcomes of these combined exposures is fundamental, as isolated laboratory assays systematically underestimate the true, synergistic risk imposed on wild and managed bee populations.

The combination of PAHs and heavy metals creates significant oxidative stress. Transition metals bypass metabolic activation to generate ROS directly via Fenton-like chemical reactions, while simultaneously binding to and paralyzing the thiol groups of essential antioxidant enzymes [138]. When a foraging bee ingests PM2.5 heavily laden with both metals and PAHs, the metal-induced suppression of the glutathione system effectively strips the cell of its primary defense mechanism exactly when the CYP450 system is generating peak levels of reactive PAH-epoxides [132,155]. This synergistic collapse of redox equilibrium guarantees that the ROS load escapes containment, inducing serious structural damage to the midgut epithelium, the primary site of both nutrient absorption and initial xenobiotic contact [136].

Pesticide co-exposure introduces a second, mechanistically distinct synergy. Sterol-biosynthesis-inhibitor fungicides such as prochloraz and tebuconazole, widely applied in fruit orchards and oilseed crops, act on bees primarily by inhibiting CYP9Q-mediated detoxification [93,156]. Because PAHs and many neonicotinoid or pyrethroid insecticides depend on the same narrow enzymatic pool for clearance, co-occurrence with SBI fungicides produces a metabolic bottleneck where parent PAHs and pesticide residues accumulate internally, mortality climbs sharply, and behavioral impairment from sublethal pesticide doses is amplified well beyond what either class produces in isolation.

The physiological toll of managing this multi-stressor oxidative burden exacts a marked energetic and immunological cost. Maintaining upregulated CYP450 and GST transcription, alongside the continuous synthesis of non-enzymatic antioxidants, forces the bee into a state of metabolic exhaustion [136]. Field assessments of bees situated in highly polluted, PAH-rich urban environments reveal elevated systemic levels of heat shock proteins (particularly Hsp70) within the intestine and fat-protein body, acting as a definitive biomarker of substantial, uncompensated cellular distress [136]. This reallocation also impairs the insect’s innate immune system. Chronic exposure to multi-stressor cocktails significantly alters the transcription of vital antimicrobial peptides, such as defensin, while depressing the phenoloxidase cascades required to encapsulate invading pathogens [136,138].

The implications of these multi-stressor interactions extend well beyond genus *Apis*. Recent controlled exposure of *B. terrestris* workers to diesel exhaust particles, a complex mixture of PAHs, ultrafine soot, and trace metals, produces not only elevated mortality but also alteration of the gut microbiome and measurable degradation of takeoff performance [23]. That such effects manifest in a wild bumble bee species under acute exposure suggests the experimental margin between laboratory toxicology and field-relevant pollutant cocktails is narrower than commonly assumed, and that managed honey bee colonies in industrialized landscapes likely sit closer to physiological tolerance limits than current regulatory frameworks acknowledge. This state of marked immunocompromise holds dire implications for colony survival. Bees suffering from PAH and metal-induced oxidative stress exhibit reduced tolerance to biological stressors [138]. Their degraded intestinal barriers and suppressed humoral defenses leave them highly vulnerable to opportunistic microsporidians, such as *N. ceranae*, and facilitate the rapid systemic replication of endemic viral loads [136]. Consequently, while environmental PAH exposure rarely results in acute, immediate mortality, its gradual disruption of the bee’s redox and immune networks significantly erodes individual longevity and overall colony resilience, accelerating the widespread pollinator declines observed in contaminated landscapes [4,86].

## 8. PFAS as Emerging Redox-Active Stressors

Per- and polyfluoroalkyl substances comprise a large and heterogeneous group of synthetic fluorinated surfactants, including legacy long-chain compounds such as perfluorooctanesulfonic acid (PFOS) and perfluorooctanoic acid (PFOA) as well as numerous short-chain and emerging alternatives. Their remarkable resistance to both biotic and abiotic degradation, along with high thermal and chemical stability, combined with frequent continuous release from industrial, firefighting, and consumer product sources, has resulted in PFAS being labeled as “forever chemicals”. Environmental monitoring shows that PFAS occur in surface and ground waters, soils, sediments, atmospheric deposition and a broad range of biota, reflecting both local emissions and long-range transport [157].

### PFAS in Honey Bees and Hive Matrices

Recent seasonal biomonitoring has demonstrated that PFAS are consistently detectable in honey bee matrices, including hive bees, foragers and collected pollen. Müller et al. [60] analyzed 90 samples from different land-use types and detected nine PFAS, with short-chain compounds such as 4:2 fluorotelomer sulfonate (4:2 FTS), perfluoropropane sulfonate (PFPS) and perfluorobutane sulfonate (PFBS) predominating. Average summed PFAS concentrations were highest in-house bees (5.29 ng g^−1^), followed by forager bees (1.93 ng g^−1^) and pollen (1.10 ng g^−1^), indicating differential accumulation and suggesting that hive bees experience greater internal exposure than foragers or pollen alone. Differences in PFAS profiles among sample types, particularly for PFPS between forager and house bees, pointed to multiple exposure routes, including ingestion of contaminated pollen and nectar, atmospheric deposition on body surfaces and contact with contaminated plant or hive surfaces.

Overall, PFAS broadly disrupt antioxidant defenses and increase ROS, causing lipid peroxidation and DNA damage across taxa, which supports a general redox imbalance/mitochondrial mechanism and justifies treating them as potential redox-active stressors in bees [157]. The first experimental data in honey bees show that chronic exposure to PFOA and perfluorohexane sulfonate at environmentally relevant residue concentrations damages the midgut epithelium, disrupts barrier integrity and alters detoxification and stress-response gene expression, implying carryover effects on brood and colony performance that warrant targeted investigation [158].

Despite emerging biomonitoring and the first experimental data, PFAS remain markedly understudied in bee redox toxicology. There are almost no standardized dose–response datasets quantifying changes in classical oxidative-stress biomarkers (SOD, CAT, GPx, GST and GSH) in honey bees under controlled PFOS, PFOA or short-chain PFAS exposure. Current PFOA research reports midgut injury together with antioxidant and detoxification responses [158]. However, it lacks a comprehensive assessment of enzymatic and non-enzymatic redox endpoints or oxidative damage markers across various bee life stages and tissues. Information on mixture toxicity involving PFAS and other stressors relevant to bees is also extremely limited. Experimental designs combining PFAS with pesticides, metals, microplastics or nutritional stress, scenarios that better reflect field conditions, have not yet been systematically developed for bees, in contrast to pesticide-pesticide mixtures where such interactions are increasingly documented [4,159]. Furthermore, environmentally realistic exposure scenarios that integrate fluctuating PFAS residues in water, nectar, pollen and air, together with colony-level dynamics and seasonal variation in foraging, are only beginning to be explored [60]. There is a clear need for systematic studies on the effects of PFAS on antioxidant enzymes, non-enzymatic antioxidants, oxidative damage markers, and microbiome-related endpoints in honey bees and wild bees, considering both single and mixed stressor conditions. Such data are essential to understand whether PFAS act primarily as independent redox-active toxicants, as modulators of other xenobiotic responses, or as subclinical co-stressors that reduce pollinator resilience to the broader contaminant mixture present in modern landscapes.

## 9. Microplastics and Nanoplastics

### 9.1. Occurrence in Bees and Hive Products

Recent field surveys have demonstrated that adult honey bees, brood and products, including honey, wax, pollen and larvae, are consistently contaminated with microplastics (MPs, <5 mm) and microfibers (MFs, diameter < 50 μm) of diverse polymer types and morphologies [160,161,162]. Using Fourier transform infrared (FTIR) microspectroscopy, Schiano et al. [160] showed that MPs and MFs are present in adult bees and honey from apiaries located in both low- and high-urbanized areas in Southern Italy, indicating that atmospheric fallout and landscape-level contamination are major sources. In their study, the majority of MFs were of natural origin, followed by synthetic fibers, and their chemical composition closely reflected textile materials, suggesting that they are released from fabrics to air and then intercepted by foragers in flight or on flowers [160]. In the same work, polytetrafluoroethylene (PTFE) MPs were identified in bees, confirming their diffuse presence as soil and air contaminants, while polyethylene (PE)-based MPs and polycaprolactone (PCL) MPs in honey were linked to the extensive agricultural use of PE films and the increasing application of biodegradable plastics in urban environments [160].

Additional studies reported that honey from different regions, including honey of stingless bees *Melipona quadrifasciata* in Brazil, systematically contains microplastics, dominated by polypropylene fibers below 300 μm and at concentrations between about 0.1 and 2.6 particles mL^−1^ [163]. In a field experiment with polyester microfibers in feeding syrup, Alma et al. [161] demonstrated that ingested fibers are incorporated into bee tissues and hive matrices and accumulate primarily in wax, whereas honey contained relatively low levels comparable to those detected in commercial products. Collectively, these findings support the concept of honey bees as “active bioindicators” or sentinels of microplastic pollution in terrestrial environments [162,164,165].

### 9.2. Effects on Gut Integrity, Microbiota and Oxidative Status

Laboratory feeding assays with spherical polystyrene microplastics (PS-MPs; ~25 μm) have shown that chronic oral exposure causes marked, often sublethal, alterations in honey bee gut physiology and microbiota [25,164]. It was reported that PS-MPs accumulate in the hindgut and rectum, where they interact with core gut symbionts and reduce bacterial α-diversity and alter community structure, while only marginally affecting body weight and survival at the tested concentrations [164]. Wang et al. [25] further showed that micro- and nano-scale PS particles induce intestinal dysplasia, structural damage to the gut wall and disruption of the intestinal barrier, accompanied by significant shifts in the composition of gut microbiota and intestinal immune signaling. At the molecular level, exposure to PS-MPs modulated the expression of genes encoding antioxidant and detoxification enzymes, including CAT, cytochrome P450s (e.g., CypQ1) and GST, as well as immune-related genes, indicating that microplastics directly impact gut redox homeostasis and local immunity [25,164]. In a complementary study was demonstrated that chronic PS-MP exposure reduces food intake, decreases survival and triggers broad metabolic reprogramming, with metabolomic profiles showing up-regulated amino acid and carbohydrate metabolism and down-regulated alpha-linolenic acid and lipid pathways, consistent with increased energetic demands for coping with oxidative damage and tissue repair [24]. Importantly, depletion of the normal gut microbiota by antibiotics markedly increased mortality in bees exposed to PS-MPs, underscoring the protective role of a balanced microbiome against plastic-induced oxidative and immune stress [25].

Emerging work on co-exposure scenarios indicates that microplastics can interact with pesticides at the level of gut integrity, microbiota and oxidative status. Mitton et al. [166] showed that combined exposure of larvae to MPs and glyphosate significantly reduced larval survival and body mass and dysregulated genes related to detoxification, antioxidant defenses and immunity, more strongly than either stressor alone. Such data highlight that MPs and nanoplastics (NPs) should be considered important co-stressors in the broader context of xenobiotic-induced oxidative stress in bees.

### 9.3. Oxidative Biomarkers and Combined Exposures

Studies targeting classical oxidative-stress biomarkers provide converging evidence that chronic exposure to MPs and NPs perturbs the antioxidant defense system in honey bees [25,164]. PS-MPs and nano-PS have been shown to alter the activities of key antioxidant enzymes, including CAT and GSTs, and to modulate the expression of multiple antioxidant and detoxification genes, often in a time-dependent pattern characterized by early compensatory up-regulation followed by depletion or inhibition under prolonged exposure [25]. In honey bee larvae, Mitton et al. [166] found that combined exposure to MPs and glyphosate significantly affected CAT activity and other oxidative biomarkers, with interaction patterns indicating that MPs can modify pesticide-induced oxidative responses during development.

When microplastics co-occur with fungicides, oxidative imbalance may be either exacerbated or partially modulated in a contaminant-specific way. In a recent study on chronic exposure, Wang et al. [167] found that difenoconazole alone disrupted the structure of the gut microbial community, leading to oxidative damage and transcriptional changes. However, when difenoconazole was co-exposed with PS-MPs, some aspects of the oxidative stress induced by difenoconazole were reduced, although this combination still increased microbial community disorder. Experiments with pharmacological and pesticidal oxidative-stress inducers in adult bees similarly revealed altered survival, protein damage and gene-regulation profiles linked to redox stress, immunity and detoxification. Tahir et al. [153] showed that exposure of newly emerged workers to paraquat, hydrogen peroxide, tunicamycin, thapsigargin, metformin and imidacloprid produced compound-specific effects on survivorship and transcriptional responses, with significant protein damage particularly after paraquat and imidacloprid exposure. These findings support the view that different xenobiotic classes can disturb antioxidant, immune and detoxification pathways in bees, although true non-additive mixture effects require experimental co-exposure designs rather than single-compound comparisons. Evidence from stingless bees also indicates that pesticides and heavy metals frequently modulate antioxidant and detoxification enzymes, including CAT, GST and SOD, and increase markers of oxidative damage such as lipid peroxidation, supporting the utility of these biomarkers as early indicators of sublethal stress across bee taxa [10].

### 9.4. Microplastics as Carriers for PFAS and Other Pollutants

MPs and NPs not only cause direct effects but also act as high surface area carriers for PFAS, PAHs and trace metals, delivering these co-sorbed xenobiotics to bees via contaminated nectar, pollen, water, plant surfaces and hive materials, and thereby intensifying local oxidative and mitochondrial stress in gut and other tissues. This vector role means that changes in antioxidant enzymes and oxidative damage markers often reflect combined actions of plastic particles and their sorbed contaminants, so risk assessment for managed and wild bees must explicitly account for MPs/NPs as multi-contaminant carriers when interpreting oxidative biomarkers and designing realistic multi-stressor experiments [167,168,169,170].

## 10. Other Emerging Contaminants and Physical Stressors

Short term exposure to ingested ZnO nanomaterials has been reported to exert neurotoxic effects in bees, with increased ROS generation and decreased mitochondrial activity indicating oxidative damage [171]. More broadly, mechanistic reviews on engineered nanomaterials show that many ENMs induce cytotoxicity via excessive ROS production and disruption of cellular redox homeostasis, suggesting a plausible hazard pathway for bees where environmental exposure occurs [172]. Data on pharmaceuticals are very limited, but veterinary acaricides used in hives can modulate detoxification enzymes and oxidative pathways, indicating that medicinal products may contribute to redox imbalance when combined with other stressors [173]. Radiofrequency electromagnetic fields (RF EMFs) have been more directly linked to oxidative stress in bees: long-term exposure to 900 MHz base station radiation caused significant changes in antioxidant biomarkers in honey bee colonies, with larvae being more sensitive than pupae [37,174]. Experimental exposure of honey bee larvae to modulated 900 MHz RF EMF (23 V m^−1^) altered GST and CAT activities and induced oxidative stress and DNA damage, while power line frequency (50 Hz) electromagnetic fields also changed SOD and CAT activities and total antioxidant capacity in adult bees [175].

## 11. Xenobiotic-Induced Changes in Antioxidant Biomarkers

### 11.1. Patterns Across Xenobiotic Classes

Across xenobiotic classes, honey bees generally show a two-phase antioxidant response: an initial increase in antioxidant and detoxification capacity at sublethal exposure, followed by loss of activity and accumulation of oxidative damage under higher or chronic doses [4,176]. In their broad review of sublethal contaminant effects, authors highlighted that enzymatic and molecular endpoints related to oxidative stress are still under-used compared with mortality or behavioral endpoints, but where measured they frequently show this compensatory-then-exhaustive pattern [4]. Among enzymatic biomarkers, SOD, CAT, GPx and GST are the most widely investigated in honey bees and consistently respond to pesticides, trace elements, and emerging contaminants [4,21,33]. Di Noi et al. [4] reported that agrochemicals and trace elements frequently modulate SOD and CAT activities, often in parallel with GST, in both laboratory and field studies. GST, CAT and SOD display predictable changes across tissues and factors, confirming their usefulness as core redox biomarkers in bees [32,177].

Pesticides are the best-documented chemical group for antioxidant disruption in honey bees. In a semi-field oral exposure study with realistic residues of acetamiprid, glyphosate and tebuconazole alone and in mixtures, authors showed that tebuconazole significantly changed several biochemical markers in hemolymph after one week, whereas mixtures produced distinct patterns compared to single substances, underscoring mixture-specific biochemical responses [104,178]. Earlier field biomonitoring by Badiou-Bénéteau et al. [51] already demonstrated that urban bees exposed to mixed pollution showed altered GST, acetylcholinesterase and alkaline phosphatase activities relative to bees from a semi-natural site, indicating combined oxidative and detoxification responses under anthropogenic pressure.

Metals and metalloids also converge on oxidative pathways in bees. A systematic review of stingless bees shows that insecticides, fungicides, herbicides and heavy metals consistently modulate antioxidant enzymes and increase markers of oxidative damage, with CAT, SOD and GST among the most responsive endpoints. Complementary laboratory and field studies in honey bees demonstrate that exposure to trace elements such as Cd, Pb and Hg alters antioxidant gene expression (e.g., SOD1, CAT) and non-enzymatic antioxidant capacity and can both activate and overwhelm redox defenses, confirming trace metals as important drivers of oxidative stress in bees [10].

MPs and NPs are emerging contaminants that increasingly show evidence of redox disturbance in honey bees. Polystyrene microplastics reduce survival, damage gut tissue, elevate oxidative stress, disrupt gut microbiota and reprogram amino acid, carbohydrate and lipid metabolism, consistent with increased energetic demand under oxidative and inflammatory stress. When polystyrene microplastics co-occur with the fungicide difenoconazole, chronic co-exposure modifies oxidative damage, antioxidant gene expression and microbiome traits in a non-additive way, indicating that MPs can act as co-stressors that reshape xenobiotic-induced oxidative responses rather than simply adding another toxic burden [24,25].

**Figure 1:**
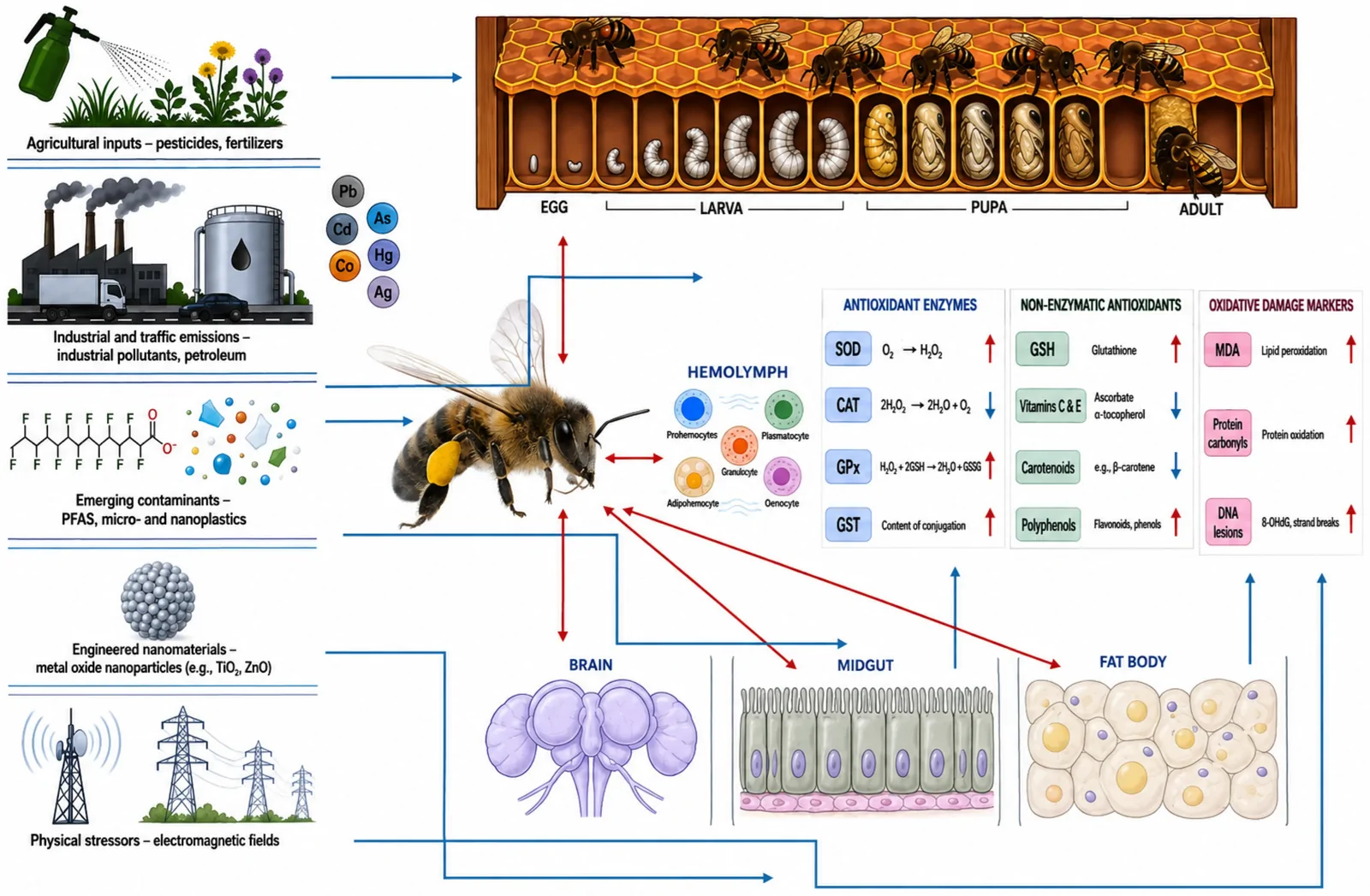
Overview of xenobiotic exposure and oxidative-stress biomarker responses in honey bee colonies.

**Figure 2:**
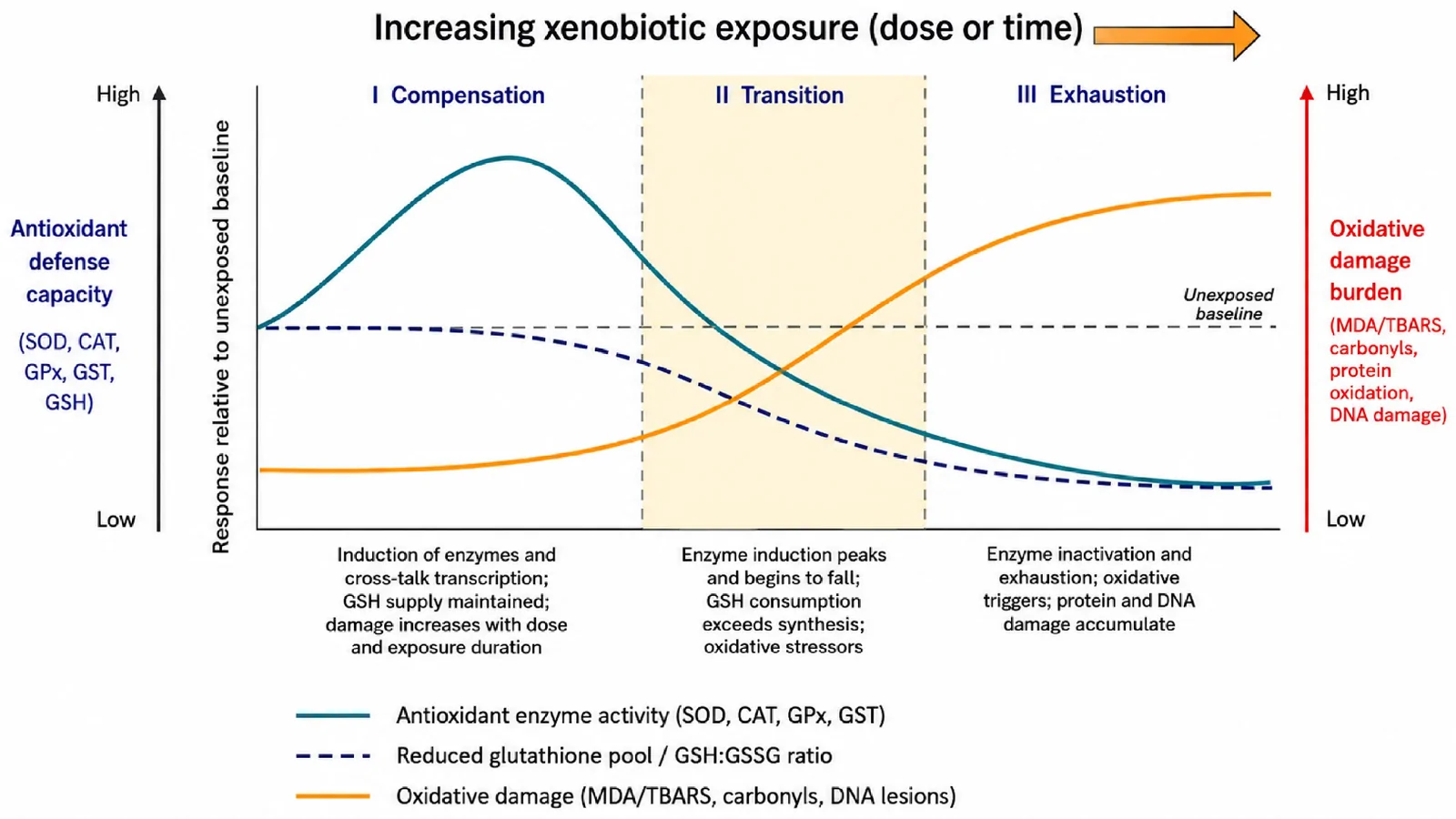
Biphasic trajectory of the bee antioxidant response with increasing dose, exposure duration, or number of co-occurring stressors. Phase I: enzyme induction with the glutathione pool largely intact and damage markers near baseline. Phase II: induction peaks and begins to fail as glutathione consumption outpaces resynthesis. Phase III: enzyme inactivation and substrate depletion, with accumulation of lipid, protein and DNA damage. Curves are schematic and are not drawn to any single dataset. CAT—catalase; GPx—glutathione peroxidase; GSH—reduced glutathione; GSSG—oxidized glutathione; GST—glutathione S-transferase; MDA—malondialdehyde; SOD—superoxide dismutase; TBARS—thiobarbituric acid reactive substances.

Markers of oxidative damage are crucial for distinguishing effective compensation from failure of the antioxidant system in bees (Figure 1). Lipid peroxidation metrics, particularly MDA, are among the most frequently applied damage markers and typically increase under pesticide, metal, or mixed contaminant exposure, while protein carbonyls and DNA lesions (e.g., comet assay; 8-OHdG) remain rarely measured despite being well established oxidative endpoints in other models [78,79,81]. The compensation-to-exhaustion trajectory that links these phases, and the biomarker signature belonging to each, is summarized in Figure 2.

### 11.2. Methodological Considerations

Interpretation of antioxidant biomarkers in honey bees depends critically on experimental design, including exposure route, dose, duration, developmental stage, caste and tissue choice. Oral exposure protocols, typically feeding bees 50% sucrose solution or sugar syrup containing dissolved pesticides, or syrup formulated to match field-realistic residue levels in honey, nectar and pollen, primarily challenge the gut epithelium and hemocoel and are most readily reflected in biochemical markers measured in hemolymph, whereas contact, fumigant or wax-associated exposures and RF-EMF act at different interfaces and can produce distinct biomarker patterns. In a laboratory assay, worker bees fed 50% sucrose containing “worst-case” environmental concentrations of acetamiprid, glyphosate and tebuconazole, alone and in mixtures, for 24 h showed significant changes in hemolymph enzyme activities and metabolites (albumin, creatinine, urea, uric acid) despite doses chosen to represent the upper range of realistic field residues [179]. Building on such work, a semi-field study exposed honey bee colonies daily for seven days to 0.5 L of sugar syrup containing acetamiprid, glyphosate and tebuconazole at concentrations corresponding to residues measured in pollen, honey and/or nectar and still detected measurable sublethal shifts in hemolymph biochemical markers, particularly for tebuconazole, underscoring that chronic low-dose exposures can modulate redox-related parameters even when doses are explicitly field-realistic [104]. In contrast, the RF-EMF study subjected colonies for one year to 900 MHz fields from base station antennas at three electric-field intensities (30, 70 and 1000 mV/m) and quantified GST, CAT, SOD and TBARS in larvae, pupae and adult worker midguts, and long-term RF-EMF exposure induced oxidative stress without a simple linear dose–response, with larvae generally more sensitive than pupae and adult midguts exhibiting the highest CAT activity and TBARS, illustrating how the nature of the stressor (chemical vs. physical), time scale and developmental stage shape redox biomarker profiles in honey bees [37,175].

Recent analyses of biomarker panels further show predictable shifts under endogenous factors such as age and caste, and document caste- and tissue-specific biomarker baselines across workers and queens, indicating that both developmental and social context must be considered when comparing antioxidant endpoints [32]. That synthesis also shows that hemolymph, midgut, fat body, brain and other organs exhibit distinct baseline and response profiles, arguing that whole-body or mixed-tissue homogenates, although practical, can mask tissue-specific effects and blur mechanistic inferences about xenobiotic metabolism and oxidative damage. Consequently, hemolymph is particularly attractive for field biomonitoring due to ease of sampling and standardization. The gut and fat body, by contrast, are often more informative for mechanistic insight into contaminant uptake, biotransformation, and redox injury, and biomarker differences across studies should not be attributed to contaminant exposure without explicit consideration of life stage, caste, and tissue context.

Current biomarker panels therefore have complementary strengths and limitations that reflect these methodological factors. A classical antioxidant damage panel comprising SOD, CAT, GPx, GST, GSH (or total thiols) and MDA/TBARS is technically accessible, facilitates comparison across studies, and has been successfully applied in field biomonitoring with honey bees as bioindicators, for example in environmental quality assessments using GST, ALP and related markers [51,178]. At the same time, recent work shows that similar redox signatures can be elicited by different contaminant classes and by non-chemical stressors, which limits causal specificity when these biomarkers are interpreted in isolation. In response, mechanistic studies increasingly combine biochemical markers with gene expression, proteomics, metabolomics and microbiome analyses to resolve pathways of action and to link oxidative stress with immune, neurobehavioral and metabolic outcomes, as highlighted in recent analyses of honey bee chemical biomarker panels and omics-based approaches [32,99]. These omics-integrated designs yield a richer mechanistic picture but reduce cross-study comparability because experimental conditions, analytical platforms and data processing pipelines differ substantially. Emerging syntheses therefore advocate a tiered strategy as a standardized core antioxidant damage panel for field biomonitoring, complemented by tissue-specific and omics-based assays in targeted experimental studies to clarify mode of action, and supported by structured data tables that explicitly record xenobiotic class, concentration, exposure route and duration, life stage or caste, tissue analyzed, markers measured, direction of effect and primary reference [32,99].

## 12. Nutritional Modulation of Antioxidant Defenses

### 12.1. Role of Pollen Diversity and Quality

Whether a colony enters winter healthy or fragile largely tracks what its foragers found within flight distance during late summer. The “landscape physiology” framework formalized this link, where bees from colonies surrounded by semi-natural habitat and floral-enriched catch crops carry measurably higher fat body mass and vitellogenin titers than those from impoverished landscapes, with these physiological signatures predicting overwinter survival better than Varroa mite load alone [180]. Pollen is not a replaceable energy source, as its botanical origin and chemical composition shape every downstream defense the bee will deploy. The baseline resilience of the bee antioxidant system fundamentally depends on the botanical origins and macronutrient balance of available pollen. Pollen supplies the essential amino acids necessary for the synthesis of immune peptides and endogenous antioxidant enzymes [11,181]. Feeding trials consistently demonstrate that pollen from insect-pollinated taxa, such as *Brassica napus*, *Phacelia*, and *Fagopyrum*, elevates SOD, CAT, and GST activities in the honey bee fat body and hemolymph more effectively than wind-pollinated sources like *Pinus* or *Corylus* [11,182]. High-quality pollen intake directly mitigates the toxic effects of agrochemicals. Exposure to acute doses of azoxystrobin and sulfoxaflor causes significantly lower mortality in bees consuming a balanced, diverse pollen diet [183].

Agricultural intensification restricts bees to homogeneous foraging environments. Monocultures often force colonies to subsist on uniform pollen diets with suboptimal protein-to-lipid ratios [184]. Restricted diets reduce physiological resilience throughout the insect’s lifespan. Worker bees deprived of adequate pollen during their larval development exhibit heightened susceptibility to clothianidin upon reaching adulthood [185]. This developmental deficit persists even if the adult bees later encounter optimal forage. Late-season access to native prairies rescues colonies from these agricultural deficits, restoring necessary lipid reserves before overwintering [186]. Winter bees must synthesize high levels of the storage protein vitellogenin to survive prolonged confinement and low temperature stress. Colonies situated near diverse conservation lands reliably produce winter bees with elevated vitellogenin titers, superior antioxidant enzyme expression, and lower winter mortality rates [187]. This nutritional foundation becomes essential when colonies face parasitic pressure. Ectoparasitic *V. destructor* mites actively deplete host protein and lipid reserves, rendering the honey bee highly vulnerable to secondary oxidative damage from viral infections and in-hive miticides [188]. High-quality pollen does not simply feed the bee, and it actively fuels the physiological barriers required to survive overlapping disease and toxicity.

These dynamics extend beyond managed honey bees to wild pollinators. Bumble bees facing identical nutritional restrictions suffer compromised immune responses and reduced lipid storage [189]. For solitary species such as *O. bicornis*, diverse pollen sources accelerate larval development and increase overwintering cocoon weight [190]. In *O. rufa*, antioxidant enzymes hold steady through diapause and then shift from fat body to hemolymph once spring flight resumes [191]. Solitary bees follow a different biochemical clock than honey bees, and feeding strategies designed for managed colonies do not necessarily transfer. Yet dietary diversity does not erase every chemical insult. A meta-analysis of solitary bee development indicates that while mixed pollen diets improve baseline physiological metrics, they often fail to buffer the developmental delays and mortality induced by field-realistic pesticide exposure [190]. This failure demands a closer examination of specific biochemical constituents within the pollen matrix. Macronutrients alone cannot fully account for xenobiotic tolerance.

### 12.2. Polyphenols and Other Dietary Antioxidants

Specific secondary metabolites from floral resources actively mediate xenobiotic detoxification and oxidative quenching, rather than acting merely as passive nutritional components. Pollen, nectar, and propolis contain specific phenolic acids and flavonoids that dictate the honey bee’s capacity to survive chemical exposure. Structurally, these compounds neutralize reactive oxygen species through direct hydrogen atom donation and metal ion chelation, preventing the initiation of lipid peroxidation cascades [12]. The flavonol quercetin and the phenolic acid p-coumaric acid, both ubiquitous in natural bee diets, upregulate the expression of cytochrome P450 monooxygenases, specifically the CYP9Q and CYP6AS subfamilies [105,192]. This enzymatic induction accelerates the metabolism of synthetic compounds. For instance, dietary p-coumaric acid increases the midgut metabolism of the organophosphate coumaphos by roughly 60%. Quercetin similarly reduces the toxicity of the pyrethroid tau-fluvalinate [193]. Following ingestion, the bee gut microbiota partially degrade and de-glycosylate these flavonoid conjugates, altering their bioactivity and facilitating their absorption into the hemolymph [194].

Evidence from multiple bioassays suggests that these phytochemicals protect against systemic neonicotinoids. The flavonoid rutin stabilizes osmotic balance and extracellular fluid dynamics in *A. mellifera* exposed to imidacloprid [195]. In the bumble bee *B. impatiens*, rutin shields individuals from imidacloprid-induced cognitive impairment, preserving motor and learning functions under exposure [196]. Other phenolic compounds offer analogous benefits. Curcumin and rosmarinic acid mitigate insecticide-induced mortality when provided as dietary supplements [197]. Extracts from propolis, particularly the bergamot polyphenolic fraction, delay mortality and reduce behavioral abnormalities in bees intoxicated by deltamethrin [198]. Even simple water-soluble antioxidants like Vitamin C, when added to feed, suppress MDA accumulation and restore glutathione peroxidase activity following peroxidative damage from imidacloprid [199].

Age and caste complicate this biochemical defense network. Antioxidant defense systems in worker bees naturally upregulate as the insect transitions from a nurse to a forager, presumably to counter the intense metabolic demands of flight. However, oral exposure to imidacloprid sharply suppresses this active defense in aging foragers, leaving them defenseless against environmental radicals. Intriguingly, older queens exhibit an opposite physiological response, and their baseline antioxidant capacity is poor, yet imidacloprid exposure triggers an immediate upregulation of their defenses, suggesting distinct evolutionary trajectories for stress management between reproductive and sterile castes [43]. Agricultural practices frequently expose bees to multi-chemical cocktails that disrupt these evolved detoxification pathways. Triazole fungicides, such as myclobutanil and propiconazole, directly inhibit the CYP450 enzymes required to metabolize quercetin [200]. When detoxification is chemically blocked, the accumulation of unmetabolized quercetin downregulates mitochondrial gene expression and suppresses ATP production. A diet rich in phytochemicals suddenly becomes a physiological liability. Consequently, exposure to seemingly benign fungicides transforms naturally occurring dietary compounds into metabolic stressors, depleting the energy reserves required for flight and foraging [200].

### 12.3. Prospects for Nutritional Interventions

Proponents of artificial diet supplementation argue that modifying commercial honey bee feed can successfully buffer the oxidative impacts of environmental contaminants. Commercial beekeepers heavily rely on soy- or yeast-based protein patties during periods of forage dearth. These standard substitutes lack the complex phenolic signatures of natural pollen [192,201]. Trace exposures to neonicotinoids interact synergistically with these simplified diets, compounding physiological stress and elevating viral titers [201]. The carbohydrate side of the substitution receives less attention. Replacing honey with sucrose syrup or high-fructose corn syrup during dearth or migratory operations strips out the p-coumaric acid and propolis-derived flavonoids such as pinocembrin and pinobanksin that upregulate detoxification and immunity gene clusters in the midgut [105]. A honey bee colony fed exclusively on syrup is, in a biochemical sense, less well defended before any pesticide arrives.

Replacing missing phytochemicals represents a logical target for feeding intervention. Recent trials substituting standard protein sources with microalgae, such as Chlorella or Spirulina, demonstrate measurable improvements in the bee metabolome and antioxidant response [202]. Formulations enriched with whey protein effectively alleviate oxidative stress induced by paraquat and manganese, restoring acetylcholinesterase and superoxide dismutase activity in exposed workers [203]. Direct supplementation with specific phytochemical extracts also yields clinical improvements in caged bee trials, often suppressing *Nosema* spp. spores proliferation alongside xenobiotic damage [11,188,204,205]. Beyond in-hive feeding, precision nutrition concepts extend to landscape management. Planting targeted floral strips adjacent to croplands could theoretically provide the exact phytochemical ratios required to rescue local pollinator populations from pesticide-induced oxidative stress [188]. Translating this concept into functional agro-environmental policy requires identifying which specific plant species deliver the optimal blend of macronutrients and protective flavonoids without harboring excessive pesticide drift from neighboring fields [206].

Pharmacological realities prohibit indiscriminate supplementation with high doses of quercetin or p-coumaric acid in commercial diets, as these compounds exhibit pronounced biphasic toxicity. While low doses promote longevity and mitigate pesticide harm, concentrations exceeding natural floral levels accelerate worker mortality [207]. Chronic supplementation overstimulates metabolic pathways, forcing a resource allocation trade-off that ultimately reduces lifespan [197].

Several practical barriers stand between phytochemical theory and applied veterinary medicine use. Our recent review [12] maps these gaps in detail: pharmacokinetic data for most plant secondary metabolites in honey bees remain sparse, with absorption, tissue distribution, and microbial biotransformation in the honey bee gut all mapped, but only in fragments. Delivery is its own problem. Isolated antioxidants frequently degrade in standard sugar syrups before bees can consume them, which is why current work has turned to nanoencapsulation and lipid carriers. Laboratory bioassays add another limitation by isolating single stressors and single phytochemicals, stripping away the multifactorial realities of the hive. Field-scale trials testing combinations of heavy metals, agrochemicals, and pathogens will determine whether targeted diets graduate into standard beekeeping practices and biosecurity measures. Until standardized formulations can mimic the safe, low-dose complexity of natural forage, preserving native floral habitats remains the most effective intervention for sustaining pollinator redox homeostasis.

## 13. From Individuals to Colonies and Populations

### 13.1. Links Between Oxidative Stress, Immunity and Disease

Xenobiotic exposure compromises hymenopteran immune competence by disrupting redox control. Pathogens that bees would otherwise carry without symptoms then escape regulation and cause overt disease. The innate immune system of the honey bee relies entirely on the maintenance of a precise redox equilibrium to regulate cellular signaling and direct antimicrobial responses [208]. When this balance collapses under the weight of pesticide exposure, the resulting physiological vulnerability offers opportunistic pathogens an open path to replication.

Converging evidence from multiple laboratory-controlled and semi-field trials demonstrates that exposure to neonicotinoids directly suppresses key immune signaling cascades. Imidacloprid and clothianidin downregulate the Toll and Imd pathways, sharply reducing the transcription of antimicrobial peptides like abaecin and defensin [15,77,153]. This chemical immunosuppression mimics the molecular damage inflicted by biological stressors. *V. destructor* parasitism alone depresses host immunity by inhibiting NF-κB activation, a fundamental transcription factor regulating the immune network [88,209]. When bees encounter both Varroa mite infestation and clothianidin, the combined stress on the NF-κB pathway triggers an unmanageable amplification of DWV [88,209,210]. The virus exploits the disabled immune barriers to replicate rapidly, converting asymptomatic covert infections into overt disease [209,210].

Theoretical modeling shows that immune suppression caused by this pathogen creates an unstable biological state, and even moderate insecticide exposure can lead to premature death in virus-infected bees [211]. A systemic viral outbreak then drives accelerated worker mortality and subsequent colony collapse [210].

Intestinal pathogens similarly exploit redox imbalance. The microsporidian *N. ceranae* fundamentally disrupts the oxidative balance within the honey bee midgut [212]. This obligate intracellular parasite imposes acute energetic stress on the host and perturbs normal antioxidant defenses, actively manipulating host ROS production to facilitate its own proliferation [208,212,213]. Co-exposure to *Nosema* spp. and sublethal doses of fipronil or imidacloprid generates pronounced synergistic toxicity, markedly increasing adult mortality rates compared to either stressor in isolation [212,213,214].

Proponents of current regulatory frameworks argue that strict pesticide limits sufficiently protect pollinators because sublethal concentrations are manageable by the insect’s baseline detoxification enzymes. This overlooks the reality of multi-stressor environments. Comparative work makes the same point at the colony level, where honey bees maintained under apiary conditions show substantially higher baseline oxidative stress and phenol oxidase activity than caged conspecifics in the laboratory, with the gap widening during *Nosema* spp. infection [177]. Laboratory exposure studies likely understate the redox load that bees face under field stressors. Environmental stressors also compromise social immunity. Worker bees use the enzyme glucose oxidase (GOX) to produce hydrogen peroxide, which sterilizes colony food and rearing environments [213,215]. While in some stationary apiaries, honey bee colonies maintain stable GOX expression, in commercial movable apiaries, colonies are subjected to chronic xenobiotic stress and heavy pathogen burdens, including the trypanosomatids *Lotmaria passim* and *Crithidia mellificae*, which display erratic GOX expression and elevated systemic MDA [215]. Systemic oxidative stress impairs the bee’s ability to melanize wounds or mount effective collective defenses. Environmental contaminants and pathogens act continuously to compound stress on these weakened bee populations [77,216].

### 13.2. Behavior, Neurotoxicity and Cognitive Functions

The disproportionately high metabolic demand of the insect brain renders it uniquely vulnerable to oxidative damage, converting xenobiotic exposure directly into behavioral and cognitive deficits. Foraging requires extraordinary spatial memory, multisensory integration, and navigational precision [77,217]. These complex cognitive processes depend on the mushroom bodies, which constitute nearly 40% of the neurons in the bumble bee brain [218]. Oxidative stress within these neural centers disrupts the foundation of resource acquisition. Neonicotinoids initiate this neurological damage by overstimulating nicotinic acetylcholine receptors (nAChRs). This continuous nervous stimulation precipitates substantial calcium influx and subsequent mitochondrial dysfunction [77,81]. As ATP synthesis falls, ROS accumulates in the neural tissue. Multiple experimental studies indicate that exposure to agents like acetamiprid and deltamethrin alters brain redox homeostasis, depleting glutathione reserves and elevating MDA levels as lipid peroxidation damages cellular membranes [13,17,81]. Heavy metals cause comparable neurological damage. Chronic exposure to Cd induces oxidative stress and reduces the expression of odorant-binding proteins in the heads of honey bee workers, leading to impaired proboscis extension response (PER) and degraded olfactory learning [102]. Airborne pollutants and extreme temperature fluctuations compound these deficits. High maximum daily temperatures positively correlate with poor air quality, directly lowering the expression of immune and buffering genes like prophenol-oxidase and Heat Shock Protein 70 (HSP70) in foraging bees [219].

These biochemical failures manifest as pronounced behavioral abnormalities. Workers exposed to sublethal doses of thiamethoxam and clothianidin exhibit reduced memory consolidation, prolonged homing flights, and outright navigational failure [15,77,154]. The disruption of memory-associated transcription factors, such as CREB and CaMKII, prevents the retention of floral cues and causes synaptic malfunction [15,77]. These cognitive impairments extend beyond managed honey bees. A controlled laboratory assessment of the bumble bee *B. terrestris* demonstrated that chronic exposure to field-realistic levels of thiamethoxam impairs short-term memory and learning speed [220].

The precise biochemical rescue mechanisms confirm the oxidative origin of these behavioral deficits. Exogenous administration of melatonin to imidacloprid-exposed bees successfully mitigates apoptosis and rescues olfactory learning by enhancing SOD and CAT activities, demonstrating that restoring redox equilibrium can preserve cognitive function [78]. Without such intervention, sublethal doses of systemic chemicals modulate mitochondrial function, generating feedback loops that directly suppress the learning and memory circuitry embedded within the antennal lobes [90]. Disoriented foragers spend more energy executing inefficient flights, gather less pollen, and fail to communicate resource locations to nestmates via the waggle dance [77]. Driven by the accumulation of ROS and the resulting apoptosis in neural tissues, this premature cognitive decline forces honey bee colonies to rely on increasingly ineffective foragers [221]. Colonies thus expend more metabolic energy to retrieve fewer resources, driving a negative feedback loop of starvation and continued oxidative deterioration.

### 13.3. Colony-Level and Population-Level Outcomes

Sublethal oxidative stress within individual workers scales non-linearly to honey bee colony collapse, as shortened lifespans and compromised queens destabilize the entire demographic structure of the hive. The survival of a eusocial insect colony relies on maintaining a precise balance between brood production and adult worker retention. When environmental xenobiotics force that system out of equilibrium, the population-level consequences are damaging. The physiological toll of oxidative stress accelerates worker senescence. Bees reacting to pesticide exposure and subsequent ROS accumulation exhibit life-shortening energetic drains [77,222].

Migratory beekeeping operations, which continuously truck hives across vast agricultural areas to provide commercial pollination, compound this exhaustion. Field experiments demonstrate that migratory adult bees suffer a significant decrease in lifespan compared to stationary colonies, driven directly by accumulated oxidative damage and diminished access to diverse forage [222]. Feral honey bee populations provide a stark contrast. Observational data indicate that foragers from feral colonies possess a significantly longer lifespan and exhibit a higher tolerance for lipid damage than their commercially managed counterparts, suggesting that natural selection has favored mechanisms of oxidative tolerance [223]. When commercially managed foragers die prematurely, the colony must artificially accelerate the behavioral maturation of younger nurse bees to replace them [185]. These precocious foragers strip the brood of necessary care and initiate a demographic decline [224,225]. A longitudinal study of pesticide mixtures indicated that while hives might withstand brief exposures, chronic dietary contact with neonicotinoids and fungicides limits adult populations and increases the risk of overwintering mortality [110,226].

Queen health dictates population stability, yet queens and workers respond differently to oxidative threats. Evolutionary pressure has shaped a distinct antioxidant phenotype in reproductive females. While aging workers experience a suppression of their antioxidant defense systems under imidacloprid exposure, older queens unexpectedly upregulate their defenses [43]. Queen reproductive physiology is itself a target. Field-realistic doses of thiamethoxam and clothianidin damage queen reproductive anatomy and reduce both the quantity and quality of spermathecal sperm. This is a one-off insult, since queens mate only briefly, which lowers colony genetic competence for the rest of their tenure [50]. Antioxidant upregulation does not extend to this reproductive damage. Chronic neonicotinoid exposure combined with parasitic infections still reduces queen survivorship and compromises fecundity. The premature loss or supersedure of a queen during periods of environmental stress frequently precedes colony failure [16,227].

Oxidative depletion becomes particularly critical during winter, when reduced cellular immune function increases the susceptibility of honey bees to DWV, especially in colonies simultaneously burdened by *V. destructor* as a viral vector [228]. Overwintering success depends strongly on vitellogenin, a nutritionally regulated storage and regulatory protein that also functions as a potent antioxidant, supporting longevity, stress resistance and the production of long-lived diutinus workers that bridge the winter and rear the first spring brood [187]. Colonies that experience poor autumn nutrition, particularly limited access to diverse, protein-rich pollen, typically show reduced vitellogenin expression and diminished antioxidant capacity and thus enter winter in a physiologically compromised state, with higher risk of mid-winter losses [187]. Targeted supplemental winter feeding can partially compensate for this deficit, and provision of artificial diet and enriched sugar syrup over extended periods enhances colony development and significantly increases SOD, CAT and glutathione levels in emerging workers, indicating that beekeeper intervention can directly modulate the antioxidant system rather than merely sustaining caloric intake [229].

Wild bees face parallel threats. *Bombus* spp. frequently ingest environmental toxicants, such as heavy metals sequestered from contaminated soils, which alter their delicate microbiome and overwhelm their oxidative stress tolerance pathways [230]. Each endpoint above, like accelerated worker senescence, queen reproductive damage, depleted vitellogenin stores, and microbiome failure in wild bees, traces back to the same biochemical bottleneck, and a colony’s capacity to mount and sustain antioxidant responses across overlapping generations and physiological states. Pesticide regulation and habitat policy ease the external load, but they relieve hives only by giving this internal chemistry room to recover; interventions that bypass it fail at the colony level even when the landscape improves.

### 13.4. Transferability of Honey Bee Mechanisms to Bumble Bees and Solitary Bees

**Figure 3:**
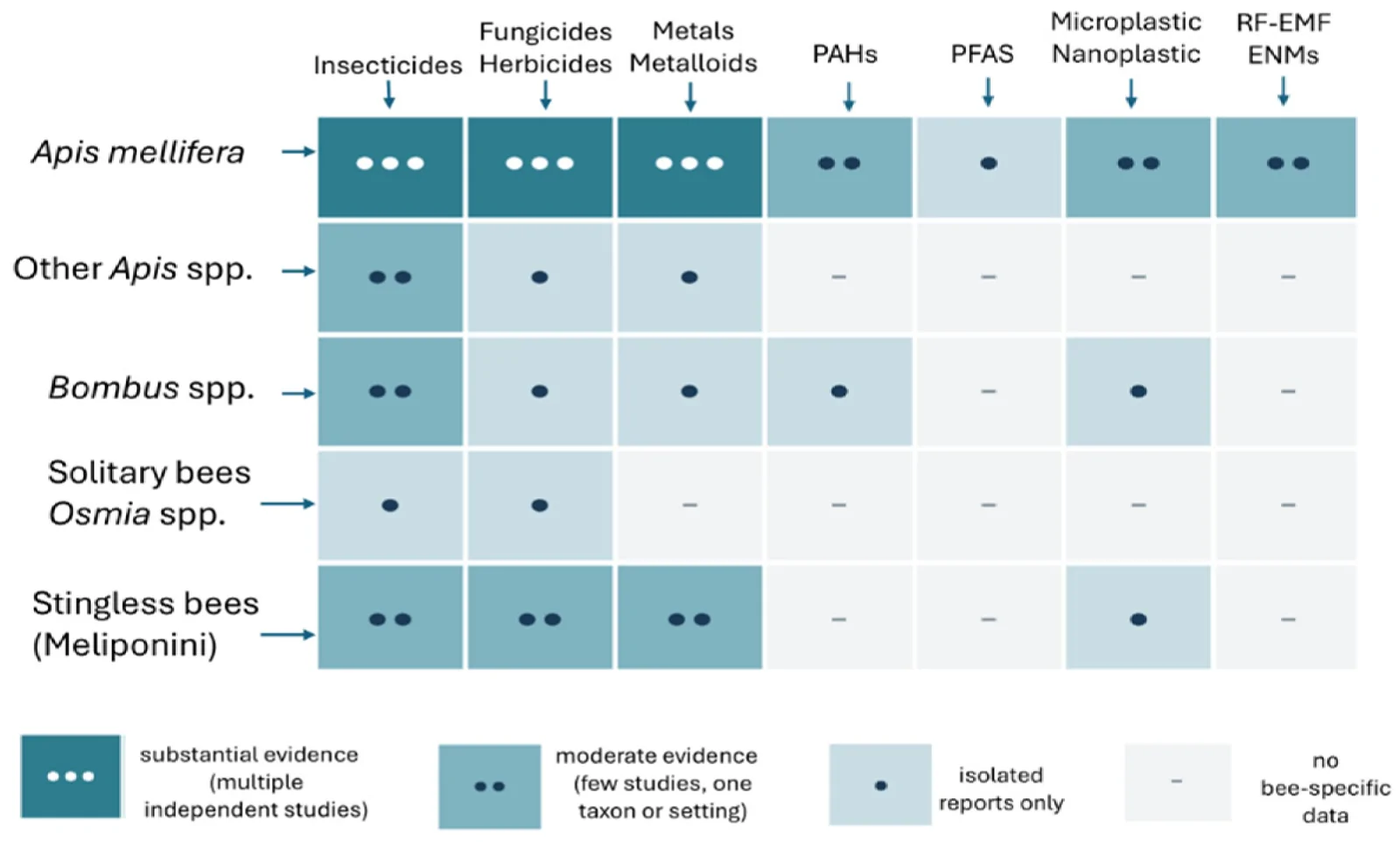
Coverage of the bee redox-toxicology literature reviewed here, by stressor class and bee taxon. ENM—engineered nanomaterial; PAH—polycyclic aromatic hydrocarbon; PFAS—per- and polyfluoroalkyl substances; RF-EMF—radiofrequency electromagnetic field.

Almost everything set out above rests on *A. mellifera*, which raises the question of how much of that account survives a change in taxon. The enzymatic core travels well. SOD, CAT, GPx and the GST family are conserved across Apidae and Megachilidae, respond to the same classes of electrophile, and are regulated through the same CncC/Keap1/ARE axis [9,34], and glutathione chemistry is not species-specific either. The qualitative expectation that a pro-oxidant xenobiotic will first induce and then exhaust these defenses is therefore a reasonable extrapolation to bumble bees and solitary bees, and the few direct tests available are consistent with it. Presentation in Figure 3 shows how quickly the supporting evidence thins once the taxon changes.

Three things do not travel. Detoxification capacity is the first. Honey bees carry a contracted CYP450 and GST repertoire [129,130], and the CYP9Q isoforms that dominate pyrethroid and N-cyanoamidine neonicotinoid clearance in *A. mellifera* have orthologues of differing catalytic efficiency in *Bombus* spp. and *Osmia* spp., so the same external dose yields a different internal dose [95,108]. Thresholds derived from honey bees cannot be read across. Colony scale is the second: a honey bee colony dilutes contaminated stores across tens of thousands of workers and absorbs forager losses, whereas a *Bombus* spp. colony of a few hundred individuals, or a solitary female provisioning her own cells, has no such buffer [26]. Life-history timing is the third. In *O. rufa*, antioxidant enzymes hold steady through diapause and then redistribute from fat body to hemolymph once spring flight resumes [191], a schedule with no honey bee equivalent; sampling protocols built around the nurse-to-forager transition will mis-time solitary bee work.

Where wild bees have been tested directly, they have not looked more resistant than honey bees. Chronic thiamethoxam at field-realistic levels impairs short-term memory and learning speed in *B. terrestris* [220], acute diesel exhaust particles shift the bumble bee gut microbiome and degrade takeoff performance [23], soil-derived heavy metals disturb *Bombus* spp. microbiota and redox tolerance [230], and stingless bees show the same modulation of CAT, SOD and GST, and then the same rise in lipid peroxidation, under pesticide and metal exposure as honey bees [10]. Pesticide and resource stress act additively on wild bee reproduction [2]. What is missing is not plausibility but calibration. Mixed pollen diets that improve baseline physiological metrics in solitary bees still fail to buffer field-realistic pesticide exposure [190], and midgut histopathology in aculeate Hymenoptera outside *Apis* spp. is documented in only a handful of species [27]. Until *Bombus* spp. and *Osmia* spp. datasets become available for the honey bee, the mechanisms reviewed here should be read as directional for wild bees and quantitative only for *A. mellifera*.

## 14. Knowledge Gaps and Future Research Directions

Several clear research gaps emerge from the current literature on redox biomarkers in bees and are highly relevant for future work. First, there is still no standardized core panel of oxidative antioxidant biomarkers that is robust across bee tissues, life stages and contaminant classes. Most studies use some combination of SOD, CAT, GST, GPx, total antioxidant capacity and GSH, but values are strongly influenced by caste, age, tissue and assay design, which makes cross-study integration and meta-analysis difficult [4,11]. Second, persistent and emerging contaminants such as PFAS and microplastics remain underexplored in bee redox toxicology, despite evidence that they occur in bees and hive matrices and can act as long-term stressors. Datasets are sparse and largely limited to screening level observations [4,60].

Third, realistic multi-stressor scenarios are rare. Experimental work still focuses mainly on single compounds, whereas bees in agricultural and urban landscapes experience complex mixtures of pesticides, metals, PFAS, microplastics, parasites and nutritional stress. Only a few studies have shown synergistic effects of pesticide and pathogen combinations on survival and immunocompetence, and comparable data for PFAS and microplastics are almost completely lacking [231,232]. Fourth, there is a persistent gap between laboratory conditions and field relevance: short, high-dose exposures are common, while chronic, low-dose, seasonally realistic and colony-level designs (especially intergenerational ones) remain the exception, even though they are most relevant for risk assessment [4,99]. Fifth, the evidence base is taxonomically lopsided. Nearly every mechanism described above was established in *A. mellifera*, and no bumble bee or solitary bee species yet has a published reference range for any redox biomarker, which prevents the calibration that risk assessment for wild pollinators would require [2,26,190].

Finally, the field still lacks integrated frameworks that routinely link biochemical redox markers to omics data, physiology, behavior and colony performance, and then connect these responses to explicit measurements of contaminants in pollen, wax, honey and other matrices. Where this has been attempted, the interpretive power of biomarkers increases dramatically, but such studies are still rare and not yet organized into coherent, comparable pipelines [11,145,157].

## 15. Conclusions

Environmental xenobiotics across multiple classes consistently disrupt antioxidant defense and induce oxidative stress in bees, with characteristic changes in SOD, CAT, GPx, GST, non-enzymatic antioxidants and oxidative-damage markers that translate into immune, behavioral and survival costs at individual and colony levels. This review shows that redox biomarkers are already powerful early-warning indicators of xenobiotic pressure, but heterogeneous assay designs, limited data for emerging pollutants and a persistent gap between laboratory toxicology and realistic multi-stressor exposure in the field constrain their interpretive value.

To move the field forward, future research should: (i) develop and validate a standardized core panel of oxidative-antioxidant biomarkers for honey bees that is robust across tissues, life stages and major contaminant classes; (ii) quantify the redox effects of PFAS and microplastics under environmentally realistic, chronic multi-stressor regimes; (iii) integrate biochemical redox markers with transcriptomic, microbiome and metabolomic datasets to connect oxidative stress with immune, behavioral and colony-level performance endpoints; and (iv) systematically couple bee-level biomarker responses with spatially explicit contaminant monitoring in hive matrices such as pollen, wax and honey to build truly field-relevant biomonitoring frameworks. Implemented together, these steps would transform antioxidant biomarkers from isolated diagnostic tools into the backbone of predictive, ecologically grounded assessments of xenobiotic impacts on bee health and pollination services, enabling mitigation at ecosystem and food-security relevant scales in line with modern bee risk-assessment guidance and One Health principles.

## Figures and Tables

**Table 1:** Main biomarkers of oxidative stress and antioxidant response in bees.

Biomarker	Significance	Method	Tissue	References
ROS generation	Oxidative load	DCFH-DA fluorescence; DHE; ESR	Hemolymph, fat body	[29]
SOD/CAT/GPx/GST activity	Antioxidant enzyme capacity	Spectrophotometric enzyme assays	All tissues; whole body	[33,35,37]
GSH-GSSG ratio	Cellular redox status	Ellman’s (DTNB); LC-MS/MS	Fat body, hemolymph	[43,44]
MDA/TBARS	Lipid peroxidation	TBA reaction; HPLC-MS	Whole body, brain	[32,37]
Protein carbonyls	Protein oxidative damage	DNPH derivatization; ELISA	Whole body, fat body	[32,35]
DNA strand breaks	DNA oxidative damage	Alkaline comet assay	Hemocytes, midgut	[32]
8-OHdG	Oxidative guanine modification	ELISA; LC-MS/MS	Brain, fat body, testes	[32]

Abbreviations: CAT—catalase; DCFH-DA—2′,7′-dichlorodihydrofluorescein diacetate; DHE—dihydroethidium; DNPH—2,4-dinitrophenylhydrazine; DTNB—5,5′-dithiobis(2-nitrobenzoic acid); ELISA—enzyme-linked immunosorbent assay; ESR—electron spin resonance; GPx—glutathione peroxidase; GSH—reduced glutathione; GSSG—oxidized glutathione; GST—glutathione S-transferase; HPLC-MS—high-performance liquid chromatography-mass spectrometry; LC-MS/MS—liquid chromatography-tandem mass spectrometry; MDA—malondialdehyde; 8-OHdG—8-hydroxy-2′-deoxyguanosine; ROS—reactive oxygen species; SOD—superoxide dismutase; TBA—thiobarbituric acid; TBARS—thiobarbituric acid reactive substances. DNA-damage endpoints are applied only rarely in bees; the sources listed for those rows are biomarker syntheses rather than primary bee studies (Section 11.1).

**Table 2:** Pesticide classes and oxidative stress biomarkers in honey bees.

Class/Examples	Main Targets (Bees)	Key Oxidative Stress Biomarkers (e.g.,)	Antioxidant/Redox Response	References
Neonicotinoids	nAChRs in the central nervous system	↑ ROS (DCFH-DA, DHE), ↑ MDA/TBARS, ↑ protein carbonyls, oxidative DNA damage, altered GSH/GSSG ratio	Early induction followed by depletion of SOD and CAT, altered GPx and GST activity, depletion of GSH, reduced total antioxidant capacity	[77,78,79,80,81]
Organophosphates and pyrethroids	Acetylcholinesterase (organophosphates); voltage-gated Na^+^ channels (pyrethroids)	↑ ROS and MDA, ↑ protein carbonyls, ↓ GSH/GSSG ratio, DNA strand breaks in hemocytes and midgut	Decreased SOD, transient CAT “spikes” followed by exhaustion, impaired GPx, strong GSH depletion and redox imbalance	[17,81,82,83,84,85]
Triazole fungicides	CYP monooxygenases, especially CYP9Q isoforms	In mixtures: ↑ ROS, ↑ MDA, ↑ protein carbonyls; more pronounced oxidative DNA damage than with insecticides alone	Overuse of SOD, CAT, GPx and GST, increased GSH consumption, prolonged insecticide half-life and cumulative redox stress	[79,92,93,94,95]
Herbicide glyphosate	Mitochondrial function and core gut microbiota	↑ mitochondrial ROS, delayed ↑ MDA and other lipid peroxidation adducts, ↑ protein and DNA oxidation under prolonged exposure	Early induction of antioxidant and mitochondrial genes, later failure to prevent oxidative injury, depletion of GSH, altered SOD/CAT/GST, weakened microbiota- mediated defenses	[90,91]
Field-realistic pesticide mixtures	Multiple targets: nAChRs, CYPs, mitochondrial complexes, ion channels	Systemic ↑ ROS, ↑ MDA, ↑ protein carbonyls, oxidative DNA damage, often non-linear vs. single compounds	Rapid GSH exhaustion, disturbed GSH/GSSG ratio, overload and decline of SOD and CAT, complex mixture-specific CYP/GST induction inhibition patterns	[62,80,96,97]

Abbreviations: CAT—catalase; CYP—cytochrome P450; DCFH-DA—2′,7′-dichlorodihydrofluorescein diacetate; DHE—dihydroethidium; GPx—glutathione peroxidase; GSH—reduced glutathione; GSSG—oxidized glutathione; GST—glutathione S-transferase; MDA—malondialdehyde; nAChR—nicotinic acetylcholine receptor; ROS—reactive oxygen species; SOD—superoxide dismutase; TBARS—thiobarbituric acid reactive substances. Arrows denote an increase (↑) or decrease (↓) relative to unexposed controls.

**Table 3:** Functional outcomes and environmental modulators of pesticide-induced oxidative stress.

Factor/Context	Main Functional Endpoints	Link to Oxidative Stress/Antioxidants	References
Neonicotinoids	Neurobehavior (olfactory learning, PER, memory, homing); lifespan; immunity	Oxidative damage in brain (↑ ROS, MDA, protein carbonyls, DNA damage) associated with learning and orientation deficits; glutathione depletion and reduced vitellogenin linked to shortened lifespan; Toll/Imd dysregulation increases *N. ceranae* and DWV burdens	[77,78,79,80,81]
Organophosphates and pyrethroids	CNS and gut integrity; development; adult robustness	ROS-driven lipid and protein oxidation and GSH/GSSG imbalance in brain and midgut coincide with impaired coordination, damaged midgut epithelium and “redox-fragile” newly emerged adults	[17,81,82,83,84,85]
Triazole fungicides + insecticides	Survival, brood production, colony fitness	CYP9Q inhibition by triazoles amplifies insecticide-induced oxidative damage (↑ MDA, protein carbonyls, DNA lesions), driving higher mortality and reduced brood and colony performance even at field-realistic exposures	[79,92,93,95,98]
Glyphosate	Metabolic resilience; foraging performance; susceptibility to co-stressors	Mitochondrial ROS and lipid peroxidation, together with dysbiosis-driven loss of microbiota-mediated antioxidant support, increase vulnerability to other pesticides and pathogens and likely accelerate senescence	[80,90]
Field-realistic mixtures	Mortality, brood rearing, overwintering, long-term colony survival	Combined pesticide cocktails cause systemic oxidative damage (lipids, proteins, DNA), rapid exhaustion of GSH and core enzymes (SOD, CAT, GPx, GST), leading to increased mortality and persistent sublethal deficits under realistic exposure regimes	[80,96,97,99]
Nutrition (polyfloral vs. monoculture)	Antioxidant capacity; detoxification; resilience to pesticides	Polyfloral, polyphenol-rich diets (p-coumaric acid, quercetin) enhance cytochrome P450 expression and antioxidant capacity, lowering ROS and MDA; monoculture diets limit these defenses and magnify pesticide-induced oxidative injuries	[12,62,100]
Abiotic co-stressors (heat, Cd, Pb)	Basal stress load; survival under exposure	Thermal stress and heavy metals raise baseline ROS and interfere with antioxidant enzymes and GSH metabolism, acting additively with pesticides to increase lipid/protein/DNA damage and further depress survival and behavior	[101,102,103]

Abbreviations: CAT—catalase; Cd—cadmium; CNS—central nervous system; CYP9Q—cytochrome P450 subfamily 9Q; DWV—deformed wing virus; GPx—glutathione peroxidase; GSH—reduced glutathione; GSSG—oxidized glutathione; GST—glutathione S-transferase; MDA—malondialdehyde; Pb—lead; PER—proboscis extension response; ROS—reactive oxygen species; SOD—superoxide dismutase.

## Data Availability

No new data were created or analyzed in this study. Data sharing is not applicable to this article.
